# Species-Specific Metabolic Identities Persist in Fabaceae Callus Cultures Under Standardized Culture Conditions

**DOI:** 10.3390/plants15152299

**Published:** 2026-07-27

**Authors:** Salma Halime, Sylvain Legay, Jenny Renaut, Cédric Jacquard, Kjell Sergeant

**Affiliations:** 1 GreenTech Innovation Centre, Environmental and Industrial Biotechnologies, Luxembourg Institute of Science and Technology, 4940 Hautcharage, Luxembourg; salma.halime@list.lu (S.H.); sylvain.legay@list.lu (S.L.); jenny.renaut@list.lu (J.R.); 2 Unité de Recherche RIBP UMR 1488, INRAE, Université de Reims Champagne-Ardenne, 51100 Reims, France; cedric.jacquard@univ-reims.fr

**Keywords:** callus culture, Fabaceae, untargeted metabolomics, isoflavonoids, saponins, somaclonal variation

## Abstract

Callus cultures derived from three species of Fabaceae were compared under identical hormonal conditions using untargeted UHPLC–MS/MS metabolomics together with phenotypic and antioxidant capacity analyses. Principal component analysis with 290 metabolites revealed species identity as the dominant determinant of the metabolic profile, indicating that species-associated metabolic signatures persist in dedifferentiated tissues. Soybean calli accumulated saponins, primarily soyasapogenol derivatives. Lupin calli were characterized by diverse isoflavones detected as aglycones, glycosylated and malonylated conjugates. Calli from the pea cultivar Karacter were dominated by hydroxycinnamate derivatives: coumaroyl methylhexose, feruloyl-coumaroyl glycoside derivatives, and caffeoyl amino acid conjugates. Within each species, the calli metabolomes were furthermore influenced by genotype, explant origin, and independent callus line establishment. Antioxidant capacity correlated with metabolite subclass composition rather than total metabolite abundance, with polyphenol-rich profiles displaying higher reducing potential than saponin-dominated metabolomes. Together, these findings provide the first systematic comparative evidence that species-specific metabolic signatures are maintained in Fabaceae callus cultures, while remaining quantitatively modulated by genotype, explant origin, and somaclonal variation. Soybean, lupin, and pea callus cultures thus provide tractable model systems for the species-specific study of respectively triterpenoid saponin, isoflavonoids, and hydroxycinnamate metabolism, offering a foundation for future biotechnological development.

## 1. Introduction

Plants produce a vast array of secondary metabolites that play essential roles in plant defence, signalling, and stress adaptation, but that also serve as valuable resources for the pharmaceutical, cosmetic, agricultural, and nutraceutical industries [1,2]. However, extracting these compounds from field-grown plants is often hindered by low and variable yields, seasonal and geographic constraints, and the risk of overharvesting [3]. As a result, developing sustainable, controllable, and environmentally friendly production platforms for high-value plant metabolites has become a major research priority [4].

Plant cell and tissue culture techniques have emerged as powerful tools for the controlled production of plant secondary metabolites [5,6]. Among these, callus culture—the proliferation of undifferentiated plant cells induced by specific auxin–cytokinin combinations—offers a robust and reproducible experimental system for studying secondary metabolism under precisely controlled conditions [7]. Yet much of the biosynthetic diversity present in the calli of agronomically important species remains poorly characterized.

Given its remarkable phytochemical diversity, the Fabaceae (legume) family represents a promising yet under-explored taxon for the study of specialized metabolism and its biotechnological exploitation. As the third largest family of flowering plants, encompassing approximately 19,500 species across 770 genera, Fabaceae are widely distributed from tropical to temperate regions [8] and exhibit a remarkable metabolic diversity. Beyond their agronomic and nutritional value, legumes produce a rich array of secondary metabolites including flavonoids, saponins, alkaloids, phenolic compounds and terpenoids [9,10,11]. Many of these compounds exhibit antioxidant, antimicrobial, anti-inflammatory, anticancer, or pesticidal activities [10,12]. This metabolic diversity is furthermore highly lineage-structured: distinct legume clades are characterized by partially non-overlapping specialized metabolite repertoires [9], making Fabaceae an ideal taxonomic framework for testing whether species-specific biosynthetic identities are retained under standardized in vitro conditions.

Within this diverse family, several economically important crop species combine high agricultural relevance with pronounced phytochemical diversity, making them attractive for in vitro metabolomic studies. Soybean (*Glycine max* (L.) Merr.), for example, is one of the world’s most widely grown legumes and a major source of plant-based protein and oil [13,14]. Soybean seeds accumulate bioactive isoflavones such as daidzein, genistein, and their malonylated and glycosylated conjugates. Furthermore, they are rich in triterpenoid saponins, namely the monodesmosidic group B and group E saponins, as well as soyasaponin DDMP derivatives. Unique to soybean are bisdesmosidic group A saponins characterized by acetylation of the terminal sugar attached to the C-22 position of soyasapogenol A [15,16]. These saponins are linked to antioxidant effects and health benefits (e.g., protection against cardiovascular disease and osteoporosis) [17]. Blue lupin (*Lupinus angustifolius* L.) is a protein-rich grain legume that has a complex isoflavonoid profile that includes the simple aglycones genistein and daidzein, as well as a distinctive set of prenylated isoflavonoids such as lupinalbin A, luteone, wighteone, and lupinisoflavones B and D. Its secondary metabolome further comprises flavones, notably chrysoeriol and its conjugated derivatives, together with triterpenoid saponins, including soyasaponins B, E, A, and DDMP. Several of these compound classes display antifungal and antioxidant activities [18,19]. Pea (*Pisum sativum* L.) is another globally important legume valued for its nutritional value. Its phytochemical profile is distinct from the previous species, characterized by hydroxycinnamic acid derivatives (including p-coumaric, ferulic, and sinapic acid conjugates), flavonoids, and phytoalexins [20]. The characteristic metabolomes of differentiated tissues, rich in saponins in soybean, prenylated isoflavonoids in lupin, and hydroxycinnamates in pea, makes these three species a particularly informative comparative framework for testing the retention of species-specific biosynthetic tendencies in dedifferentiated tissues.

Despite the increasing availability of metabolomic data for Fabaceae tissues, the metabolome of in vitro cultured dedifferentiated plant cells is insufficiently characterized. Untargeted metabolomic profiles generated from in vitro systems are strongly influenced by species, genotype, explant origin, and culture conditions [21]. Callus cultures, which provide a representative baseline for downstream suspension cultures, constitute a logical starting point for untargeted metabolomic characterization under controlled conditions [22]. This is all the more the case since it is unknown to what extent dedifferentiation preserves or reprograms the specialized metabolic identity of source tissues in Fabaceae. Callus formation is frequently associated with substantial transcriptional and metabolic reorganization during cellular dedifferentiation [22], raising the possibility that specialized metabolic profiles may be partially reset in vitro. This question is particularly relevant for legumes, which exhibit pronounced lineage-specific variation in alkaloid, saponin, and flavonoid composition [18,20,23]. Yet whether these lineage-specific metabolic profiles are retained in dedifferentiated tissues has not been systematically examined.

In this study, we investigate the untargeted secondary metabolite profiles of callus cultures derived from three important Fabaceae species: two soybean cultivars (Nessie and Tofina), blue lupin (Bolero), and pea (Karacter), established from two explant types (cotyledon and stem) under identical hormonal conditions. Our objectives are to (i) characterize and compare the untargeted metabolite profiles of these callus cultures across species, cultivars, and explant origins, and evaluate whether species-level biosynthetic identities are preserved following cellular dedifferentiation; (ii) examine the contribution of genotype, explant origin, and independent line establishment to intraspecific metabolic variation; and (iii) evaluate antioxidant capacity (ferric reducing capacity) in relation to metabolomic composition to assess the functional consequences of pathway-level metabolic differences.

## 2. Results

### 2.1. Comparison of Callus Fresh and Dry Weight Across Species, Cultivars, and Explant Types

Callus induction was evaluated in five Fabaceae species—*L. angustifolius* cv. Bolero, *P. sativum* cvs. Karacter and Greenway, *Vicia faba* cvs. Magnolia and Jasmin, *Cicer arietinum* cvs. Elmo and Orion, and *G. max* cvs. Nessie and Tofina—using cotyledon and epicotyl explants. For each treatment, ten independent explants were cultured to determine induction efficiency. Sixty days after callus induction, in a subculture cycle, the growth of the calli was evaluated (Supplemental Table S1) and 11 calli that showed proliferation selected for further study (Table 1). Although the addition of more calli would have been preferred, and the induction of calli was repeated to provide these, this set of calli allows to answer the scientific question asked.

Cotyledon-derived explants of Bolero and Karacter exhibited 100% induction efficiency within three weeks, whereas Nessie cotyledons formed friable calli in approximately 50% of explants. In contrast, cotyledon explants of Tofina did not produce any callus under the tested conditions. The complete absence of callus induction from Tofina cotyledon explants, suggests genotype- and explant-specific hormonal sensitivity to the applied auxin–cytokinin treatment. Karacter cotyledon-derived calli, although successfully induced, failed to proliferate upon subculture and were discarded from the experiment. Epicotyl explants of Bolero produced calli that initially appeared white but progressively developed dark brown pigmentation following subculture, accompanied by reduced growth. Karacter epicotyl-derived calli similarly acquired reddish-brown coloration and became compact and nodular over time. In contrast, soybean epicotyl explants showed limited initial callus formation, but cells regained viability and growth following transfer to fresh medium (Supplementary Figure S1).

Biomass yield differed significantly among species, cultivars, and explant types (Table 1). Biomass values represent independent biological replicates (*n* = 3) and are expressed as mean ± SD. Statistical analysis was performed using one-way ANOVA, considering the independent callus line as the explanatory factor, followed by Tukey’s post hoc test for pairwise comparisons (*p* < 0.05). Cotyledon-derived Bolero calli achieved the highest fresh weight (4.93 ± 2.37 g), although variability among replicates reflected heterogeneous growth dynamics among independently induced explants. Epicotyl-derived Bolero calli accumulated lower biomass. In soybean, Tofina calli produced greater biomass than Nessie, with epicotyl-derived fresh weights ranging from 2.26 ± 0.46 to 3.01g ± 0.07 and corresponding dry weights from 0.17 ± 0.02 to 0.21g ± 0.01. Nessie cotyledon-derived calli showed comparable fresh biomass but slightly lower dry weight. Karacter calli exhibited the lowest overall biomass, with fresh weight around 1.13 g and dry weight approximately 0.09 g.

### 2.2. Antioxidant Activity (Ferric Reducing Capacity: FRAP) Comparison Across Callus Lines

**Figure 1:**
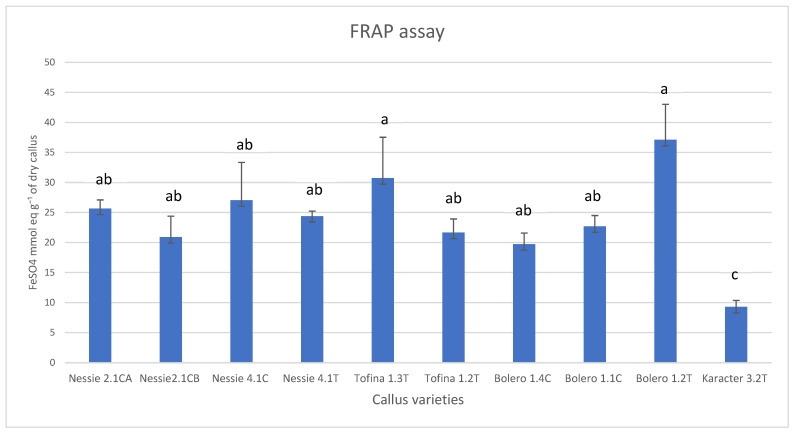
Ferric reducing capacity of calli extracts from Fabaceae species measured by the FRAP assay. Statistical differences between groups were analyzed using Tukey’s post hoc test following one-way ANOVA, performed in OriginPro 2024. Data are presented as mean ± standard deviation (SD) (*n* = 3), with significant differences indicated by different letters (*p* < 0.05).

Antioxidant capacity, measured using the FRAP assay, varied among species, cultivars, and explant types (Figure 1), although significant differences are few. Among soybean calli, the Tofina epicotyl-derived line 1.3T exhibited the highest antioxidant activity, reaching approximately 30 µmol FeSO_4_ equivalents g^−1^ dry callus. The other soybean calli showed an antioxidant capacity ranging from approximately 20 to 27 µmol FeSO_4_ equivalents g^−1^ dw, with no significant difference between cotyledon- and epicotyl-derived calli. In Bolero, antioxidant activity was variable depending on the explant origin. While cotyledon-derived lines (1.4C and 1.1C) exhibited moderate FRAP values (approximately 19–23 µmol FeSO_4_ equivalents g^−1^ dw), the epicotyl-derived line 1.2T showed the highest antioxidant capacity among all samples, reaching nearly 37 µmol FeSO_4_ equivalents g^−1^ dry callus.

In contrast, calli for the pea cultivar Karacter had the lowest ferric reducing capacity of all, indicating limited reducing capacity under the tested conditions. Overall, ferric reducing capacity differed among species and explant types, with epicotyl-derived calli from Bolero and Tofina exhibiting the highest reducing potential, whereas Karacter calli showed significantly lower activity. These values are considerably lower than FRAP values reported for differentiated Fabaceae tissues, including pea seeds (740–840 µmol FeSO_4_ g^−1^ dw) and methanolic extracts of wild and cultivated Fabaceae plants (110–1200 µmol FeSO_4_ g^−1^ dw), consistent with the reduced accumulation of free phenolic compounds typically observed in dedifferentiated callus tissue relative to intact plant organs.

### 2.3. Global Overview of Untargeted Metabolomic Profiles and Sample Clustering

Callus samples were analyzed using UHPLC–MS/MS in both ionization modes, representative LC-chromatograms are shown in Supplemental Figure S2. Raw data were processed using Progenesis QI (v2.3, Nonlinear Dynamics, Waters, Newcastle, UK) for peak detection, alignment, and normalization. The resulting feature matrix comprised all detected ion features across samples prior to metabolite annotation.

**Figure 2:**
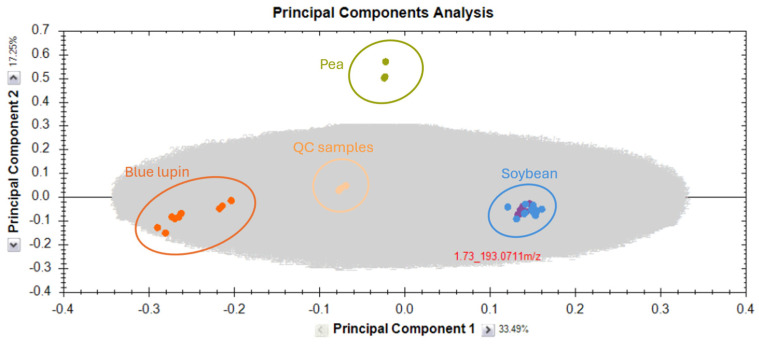
Principal component analysis (PCA) of global UHPLC–MS/MS feature profiles from Fabaceae callus cultures. PCA was performed on the complete, non-filtered feature dataset obtained after peak detection and alignment in Progenesis QI. The first principal component (PC1) explained 33.49% of the total variance, while the second principal component (PC2) accounted for 17.25%. Quality control (QC) samples, prepared by pooling equal aliquots of all samples and injected before, between, and after the analytical sequence, clustered tightly at the centre of the PCA plot. Soybean samples were represented by two cultivars, Nessie (blue) and Tofina (purple), which clustered closely together.

To assess overall metabolic variability, principal component analysis (PCA) was performed on the complete feature dataset (Figure 2). PCA revealed clear clustering according to species, indicating distinct metabolic profiles based on species. The first principal component (PC1) explained 33.49% of the total variance, while PC2 accounted for 17.25%, together capturing 50.74% of the dataset variability. Separation along PC1 primarily distinguished soybean and blue lupin samples, whereas PC2 contributed to the separation of pea from the other two species. Replicates clustered tightly, indicating good analytical reproducibility and limited intra-group dispersion.

### 2.4. Annotation of Differential Metabolites by UHPLC–MS/MS

Following this global overview, features were filtered to identify those exhibiting substantial intergroup differences (fold change > 2; *p* < 0.05). The selected discriminant features were subsequently subjected to MS/MS-based structural elucidation and database matching, resulting in the tentative annotation of 290 compounds following removal of redundant features, including adducts and dimers. Metabolite annotation was performed using both negative and positive electrospray ionization (ESI) modes, with the negative mode used as the primary ionization mode and the positive mode employed to confirm structural assignments. Unless otherwise stated, all *m*/*z* values and fragment ions reported in the following sections correspond to negative ESI mode data. A comprehensive list of the annotated metabolites, including tentative identification, *m*/*z* values of precursor and fragments, and retention times, is provided in Supplementary Table S2.

The curated metabolomic dataset was dominated by specialized metabolites, primarily flavonoids (124 compounds). Of these, 88 were classified as isoflavonoids, generally detected in multiple structural forms: free aglycones, glycosylated derivatives, glycosylated–malonylated conjugates, and hexosyl-coumaroyl derivatives. The following isoflavone aglycones were identified: daidzein, luteone, biochanin A, formononetin, and lupinisoflavones B and D.

**Figure 3:**
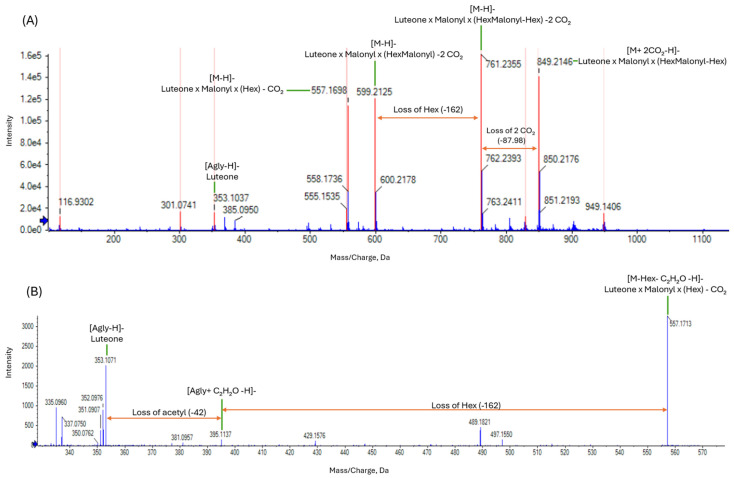
Mass spectrometric characterization of dimalonylated luteone dihexoside (compound 220) in negative ESI mode. (**A**) Negative ESI-MS spectrum showing the deprotonated molecular ion [M − H]^−^ at *m*/*z* 761.23 and prominent in-source fragment ions. (**B**) MS/MS fragmentation spectrum of the in-source fragment ion at *m*/*z* 557.17, displaying diagnostic product ions including the luteone aglycone at *m*/*z* 353.10, neutral loss of hexose (−162 Da), and the acetyl-aglycone indicating that one of the malonyl groups could be attached to the aglycone.

Figure 3 illustrates the structural elucidation of a dimalonylated luteone dihexoside (compound 220) using negative and positive electrospray ionization tandem mass spectrometry (ESI–MS/MS). In the negative ionization mode, MS analysis reveals two dominant [M − H]^−^ ions at 849.22 and 761.24 (Figure 3A). The mass difference of 87.98, and a smaller peak at 805.22, show the double loss of CO_2_, which, based on the known prevalence of malonylations and the dominant in-source loss of CO_2_ in negative mode MS from malonylated compounds, indicates that two malonyl groups are present. The presence of a single ion at *m*/*z* 851.37 in positive mode MS confirms that this may indeed be the case. The in-source fragment at 557.17, showing an MS/MS spectrum similar to that of 849 and 761, corresponds to a loss of these two molecules of CO_2_ and a hexose. In the MS/MS spectrum of the latter in-source fragment (Figure 3B) the subsequent loss of acetyl-hexose (204.06, malonyl hexose—CO_2_) to the aglycone at 353.10 is observed. A low intensity fragment ion at *m*/*z* 395.11, corresponds to the aglycone with an acetyl group attached, indicating that one of the malonyl groups may be attached to the aglycone. In the fragmentation spectrum of the compound at *m*/*z* 761.24, the same addition of 42.01 is also observed from the fragment at 557.17, indicating that the second malonyl is attached to the proximal hexose. The aglycone was identified based on MS/MS similarity in the positive and negative modes—for instance, the dominance of a fragment at 299.05 in positive mode MS/MS, as was previously observed for luteone.

Additional compounds identified that belong to different flavonoid subclasses comprised 14 flavones, 10 flavonols, 8 flavanonols, and 2 flavanones. Flavones were mainly represented by tricin, chrysoeriol, and apigenin derivatives, while flavonols consisted predominantly of morin and its conjugated forms. Flavanonols were primarily dihydrokaempferol derivatives, and flavanones included two naringenin isomers. Overall, most compounds occurred as glycosylated and malonylated derivatives, highlighting the structural diversity within these subclasses.

Hydroxycinnamic acids and their conjugates represented the second important metabolite class (58 compounds). Identified hydroxycinnamates included *p*-coumaric, caffeic, ferulic, and sinapic acids, detected either as free compounds (confirmed with standards) or as conjugated forms. These comprised glycosylated, malonylated, and derivatives with the amino acids aspartic and glutamic acid.

Saponins constituted the third major metabolite class (38 compounds) and included group A, B, E, and DDMP saponins, together with multiple glycosylated triterpenoid aglycones such as hederagenin, medicagenic acid, and olean-12-ene-3β,24-diol. Glycosides of aglycone B and aglycone D, were also tentatively identified. These saponins exhibited diverse glycosylation patterns composed of hexose (Hex), deoxyhexose (dHex), pentose (Pent), and hexuronic acid (HexA) residues in mono- or multi-saccharide chains. In several cases, sugar residues were further acylated by malonyl groups, indicating additional structural diversification. Combinatorial annotations (e.g., Hex–HexA or dHex–Hex–HexA) reflect the sequential attachment of sugar units to the triterpenoid aglycone core. Lipids accounted for 17 metabolites and were primarily represented by glycerophospholipids, hydroxy fatty acid amides, glucuronosylated lipids, and monoacylglycerols. The remaining tentatively identified compounds are sugars, nucleotides, and other primary metabolites.

### 2.5. Cross-Species Metabolomic Comparison Reveals Species-Specific Secondary Metabolite Profiles

Principal component analysis (PCA) with the 290 curated metabolites confirmed interspecific metabolic differentiation (Supplementary Figure S3). Hierarchical clustering analysis (HCA) of the same metabolite matrix resolved the samples into three primary branches corresponding to the three species (Supplementary Figure S4). Within the soybean branch, the two cultivars, Nessie and Tofina, formed two distinct and internally coherent subclusters, indicating consistent cultivar-level metabolic differentiation superimposed on species identity. At the metabolite level, the heatmap revealed structured clusters of co-abundant compounds displaying species-enriched patterns (Supplementary Figure S4). Discrete metabolite subsets characterized Bolero and Karacter, whereas soybean samples exhibited broader but still cultivar-specific abundance signatures. Within Bolero, three distinct blocks of metabolites corresponded to line-specific enrichment patterns, highlighting additional line-level divergence.

To verify that the observed species-level separation reflects species-specific specialized metabolism rather than general physiological differences, PCA was repeated on the specialized metabolite subset only (isoflavonoids, flavonoids, hydroxycinnamic acid derivatives, saponins, and related conjugates), excluding primary metabolites (sugars, organic acids, amino acids, nucleotides, and lipids). The species-level clustering observed for the full dataset was fully recapitulated and even strengthened—with PC1 alone explaining 41.7% of the variance (Supplementary Figure S5)—and was supported by hierarchical clustering analysis on the same subset (Supplementary Figure S6). Compound-class-specific analyses further showed that saponins (Supplementary Figures S7 and S8), flavonoids/isoflavonoids (Supplementary Figures S9 and S10), and hydroxycinnamic acid derivatives (Supplementary Figures S11 and S12) each independently structure samples according to species, confirming that the species-specific structure of the callus metabolome is driven by specialized biosynthetic pathways. It is worth noting that the strength of species-level clustering differed between compound classes: saponins and flavonoid/isoflavonoid subsets each showed strong species-specific clustering with high PC1 variance (50.3% and 47.6%, respectively), while the hydroxycinnamic acid subset displayed weaker but still clearly detectable species-level structuring (PC1 = 29.6%), reflecting the more universal occurrence and physiological plasticity of phenylpropanoid pathway intermediates compared with the more lineage-specific saponin and flavonoid/isoflavonoid classes. PCA on the primary metabolite subset alone (Supplementary Figures S13 and S14) also resulted in species-level grouping but with greater within-species dispersion and a partial separation between cultivars, indicating that primary metabolites capture physiological differences in addition to species-level distinctions.

**Figure 4:**
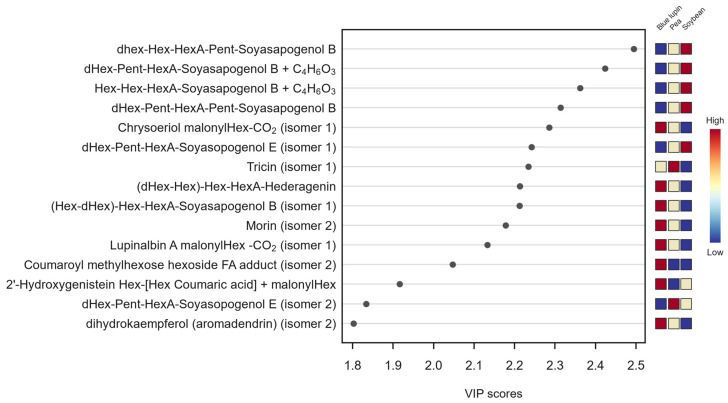
VIP score plot from PLS-DA showing metabolites contributing to interspecific discrimination among blue lupin, pea, and soybean callus cultures. Metabolites with the highest VIP values were predominantly glycosylated triterpenoid saponins and flavonoid/phenylpropanoid derivatives. The adjacent heatmap indicates scaled relative abundance across species (red = higher, blue = lower).

To identify metabolite-driving interspecific discrimination, one-way ANOVA with FDR correction was applied to the 290 annotated metabolites, revealing extensive divergence across major secondary metabolite families. In parallel, multivariate analysis using PLS-DA supported these patterns and highlighted the variables contributing most strongly to species separation. The PLS-DA model showed strong cross-validation performance (R^2^ = 0.998, Q^2^ = 0.987, 100% classification accuracy at 3 LVs; Supplementary Figure S15) with a borderline permutation test result (1000 permutations, *p* = 0.054; Supplementary Figure S16). PLS-DA and VIP scores are therefore interpreted as complementary to the unsupervised PCA and HCA analyses. Saponins with soyasapogenol B and E aglycones were among the most discriminant metabolites, being consistently enriched in soybean and showing high VIP scores (Figure 4). Blue lupin was characterized by elevated levels of lupin-associated flavonoid and isoflavone derivatives, including lupinalbin A, morin conjugates, chrysoeriol derivatives, lupinisoflavone B/D glycosides, and saponins with hederagenin and soyasapogenol B aglycones. In contrast, pea displayed relative enrichment of hydroxycinnamate conjugates and specific flavonoids, including coumaroyl methylhexose derivatives, coumaroyl–hexoside conjugates, and specific tricin and biochanin A isomers. Different daidzein and coumestrol derivatives showed highly significant interspecific differences (F values > 400; FDR < 10^−18^). Collectively, the differential accumulation of triterpenoid saponins in soybean, flavonoid/isoflavone derivatives in lupin, and hydroxycinnamate conjugates in pea accounted for the species-level separation.

### 2.6. Intraspecific Metabolomic Variation

To further investigate genotype and explant-dependent variation within species, metabolomic profiles were analyzed separately for soybean and lupin callus lines. In contrast, pea (Karacter) was not included in the intraspecific analysis, as only a single callus line was available. The Karacter line was therefore used exclusively to illustrate interspecific metabolic variation in the global comparisons but did not allow assessment of genotype or explant-dependent divergence.

#### 2.6.1. Soybean (Glycine Max)

- Cultivar effect: metabolic divergence between Nessie and Tofina

PCA with the complete feature dataset primarily separated samples according to species (Figure 2), soybean cultivars (Nessie and Tofina) largely overlapped in the multivariate space, indicating limited cultivar separation at the global metabolic level. Therefore, targeted univariate comparison between the cultivars Nessie and Tofina calli was performed on the curated set of annotated metabolites. In total, 109 metabolites showed significant differential relative abundance following multiple testing correction (adjusted *p* < 0.05), with 72 enriched in Nessie and 37 enriched in Tofina. The discriminant features were predominantly specialized metabolite conjugates, particularly isoflavonoids, saponins, and hydroxycinnamic acid-derived compounds.

Nessie calli were characterized by elevated levels of multiple isoflavonoid derivatives, with formononetin among the most discriminant metabolites (log_2_FC up to 4.96). Formononetin was detected in the negative ionization mode at *m*/*z* 267.06 (RT 26.17 min) (compounds 236, 187, 207). The MS/MS spectrum displayed characteristic fragment ions at *m*/*z* 252.03 (−CH_3_), 251.03, 223.03, and 195.04. The detection of multiple formononetin isomers further supports cultivar differences not only in isoflavonoid abundance but also in conjugation/isomeric composition. In addition to isoflavonoids, Nessie accumulated higher levels of a saponin tentatively annotated as Hex–HexA–soyasapogenol B + C_4_H_6_O_3_ (RT 35.05 min) (compound 265).

In contrast, Tofina calli exhibited a higher relative abundance of hydroxycinnamates. Sinapic acid was identified as a representative discriminant metabolite and detected in the negative ion mode as [M − H]^−^ at *m*/*z* 223.1 (RT 21.34 min) (compound 104), with identification confirmed by matching retention time and MS/MS to a standard. In addition to free sinapic acid, Tofina accumulated several conjugated hydroxycinnamate derivatives, including feruloyl dihexoside, multiple HMG-ethyl hexoside derivatives, and coumestan glycosides, all of which showed significantly higher relative abundance than in Nessie (adjusted *p* < 0.05).

Overall, these data indicate that even under identical culture conditions, calli from different soybean genotypes show distinct quantitative biases in secondary metabolism. Calli generated from the genotype Nessie had a higher accumulation of certain isoflavonoids and soyasapogenol-type saponin derivatives, whereas coumestan derivatives—as well as free and conjugated hydroxycinnamate metabolites—accumulate to higher levels in Tofina calli.

- Explant effect in Nessie: epicotyl- versus cotyledon-derived calli

Within Nessie, PCA separates epicotyl-derived from cotyledon-derived calli along PC1 (24.9% variance), demonstrating an explant effect. Univariate analysis identified 56 significantly different metabolites between calli from either explant type (adjusted *p* < 0.05), with 38 enriched in epicotyl-derived calli and 18 enriched in cotyledon-derived calli.

Epicotyl-derived calli accumulated higher levels of saponins, including Hex-HexA-Soyasapogenol E (log_2_FC = 4.99), dHex-Pent-HexA-Soyasapogenol E (log_2_FC = 4.57), and Hex-HexA-Soyasapogenol B + C_4_H_6_O_3_ (log_2_FC = 4.21). Multiple additional soyasapogenol A/B glycosides carrying combinations of Hex, dHex, Pent, and HexA saccharides were enriched (log_2_FC 1.6–3.8). Similarly, glycosylated hydroxycinnamic acid conjugates such as Coumaroyl-Hex-Coumaroyl Hex Hex + gallic acid (log_2_FC = 5.81) and Feruloyl-Hex-Coumaroyl malonylHex Hex (log_2_FC = 4.38) were increased in epicotyl-derived cultures. Whereas cotyledon-derived calli were relatively enriched in simpler hydroxycinnamate and flavonoid derivatives, including p-coumaric acid (log_2_FC = −2.93), Feruloylglucose (log_2_FC = −1.70), Morin hexoside isomers, and Daidzein hexoside. These findings demonstrate that explant origin influences the metabolic profile of calli generated from the same genotype.

- Independent line divergence in Tofina epicotyl-derived calli

**Figure 5:**
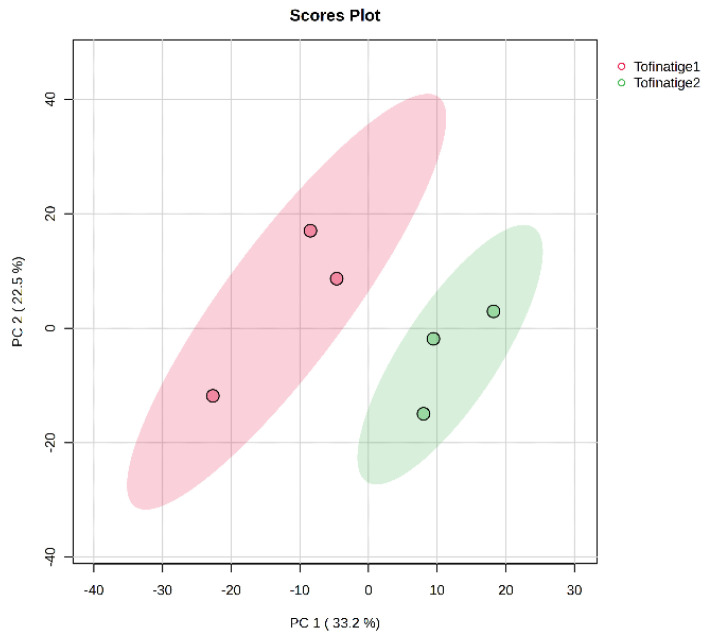
Principal component analysis (PCA) score plot of two independently established epicotyl-derived callus lines of soybean cv. Tofina. PC1 and PC2 explain 33.2% and 22.5% of the total variance, respectively. Each point represents one biological replicate. Ellipses indicate 95% confidence intervals of the group centroids.

Although both Tofina callus lines originated from epicotyl explants of the same donor plant, PCA demonstrated separation along PC1 (33.2%) and PC2 (22.5%), indicating divergence between calli independently established from the same plant (Figure 5).

A total of 35 metabolites were significantly different (adjusted *p* < 0.05). One line displayed strong enrichment of saponins, most prominently Hex-Hex × dHex-HexA-Soyasapogenol A (log_2_FC = 18.19), alongside multiple soyasapogenol B and E derivatives (log_2_FC 1.2–3.8). This saponin-dominant profile was accompanied by increased levels of sinapoylglucose (log_2_FC = 6.30) and HMG ethyl hexoside (log_2_FC = 3.73).

In contrast, the second line accumulated higher levels of free hydroxycinnamic acids and specific flavonoids, including sinapic acid (log_2_FC = −16.69), Coumaroyl aspartic acid (log_2_FC = −5.43), and Apigenin malonylHex (log_2_FC = −2.21).

These results demonstrate that, in Tofina, independently established callus lines undergo distinct metabolic reprogramming trajectories despite identical genotype and explant origin, particularly affecting triterpenoid glycosylation and phenylpropanoid conjugation patterns.

#### 2.6.2. Lupin (LUPINUS Angustifolius)

- Explant-dependent metabolic variation (cotyledon vs. epicotyl)

**Figure 6:**
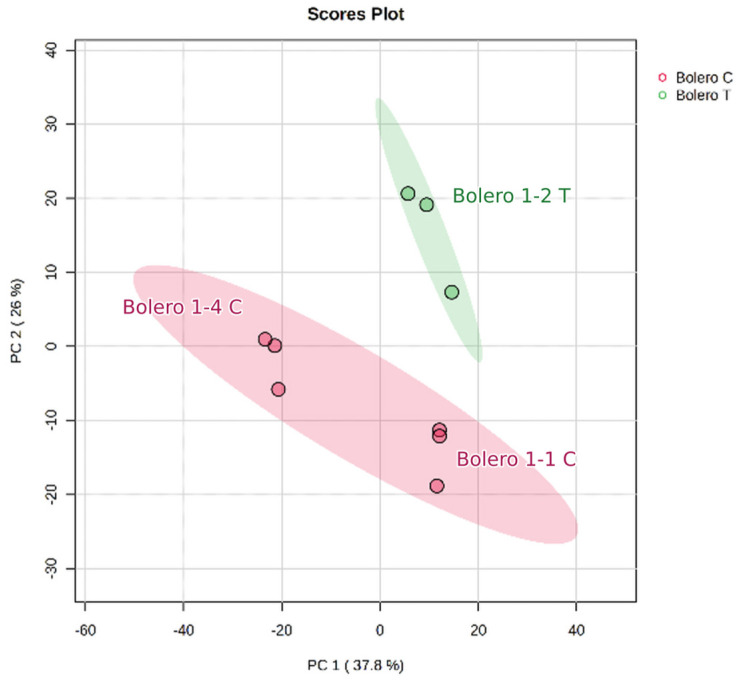
Principal component analysis (PCA) of Bolero cotyledon- (Bolero C) and epicotyl-derived (Bolero T) callus lines. PC1 and PC2 explain 37.8% and 26.0% of the total variance, respectively.

In *L. angustifolius* (Bolero), comparison of the two cotyledon-derived callus lines (Bolero 4-1C and Bolero 1-1C) and the epicotyl-derived line (Bolero 1-2T)—all originating from the same donor plant—revealed line-specific metabolic divergence. PCA performed on the annotated metabolite dataset demonstrated clear separation among the three callus lines. The first two principal components explained 63.8% of the total variance (PC1: 37.8%; PC2: 26.0%). Separation along PC1 distinguished one cotyledon-derived line (Bolero 1-4C) from the remaining samples, while PC2 further resolved the three lines from each other in multivariate space (Figure 6). Importantly, the observed clustering pattern did not correspond strictly to explant origin, indicating that metabolic differentiation was primarily line-dependent rather than explant-dependent, despite identical genetic background.

**Figure 7:**
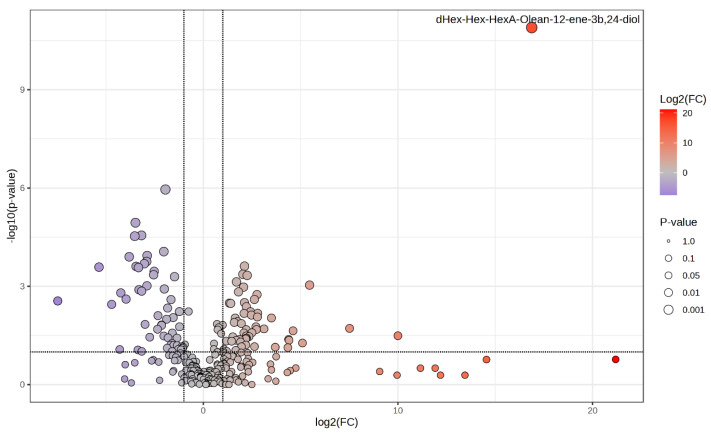
Volcano plot illustrating differential metabolite abundance between cotyledon-derived and epicotyl-derived callus lines of *L. angustifolius* cv. Bolero. The X-axis represents Log2 fold change (Log_2_FC) and the Y-axis represents −log10 (*p*-value). Red points indicate metabolites enriched in cotyledon-derived calli, blue points indicate metabolites enriched in epicotyl-derived calli, and colour intensity reflects the magnitude of Log_2_FC. Point size is proportional to statistical significance. Vertical dotted lines indicate Log_2_FC thresholds of −1 and +1, and the horizontal dotted line indicates the significance threshold (*p* = 0.05). The most discriminant metabolite, dHex-Hex-HexA-Olean-12-ene-3β,24-diol, is labelled.

Univariate analysis identified extensive metabolic remodelling, with 57 features that are more abundant in cotyledon-derived calli and 53 in epicotyl-derived calli (adjusted *p* < 0.05). Most differences were associated with saponins, lignans, hydroxycinnamic acid conjugates, and lipid-related metabolites (Figure 7).

Cotyledon-derived calli were enriched in saponins, isoflavonoids, and hydroxycinnamic acid derivatives. The most discriminant metabolite was dHex-Hex-HexA-Olean-12-ene-3β,24-diol (log_2_FC = 16.87, *p* = 1.26 × 10^−11^), accompanied by multiple glycosylated soyasapogenol B and E derivatives (log_2_FC = 1.70–4.34) and bayogenin and hederagenin conjugates. Several isoflavonoid derivatives were also elevated, including lupinisoflavone B/D malonylhexosides (log_2_FC = 2.50–4.41), daidzein isomers (log_2_FC = 2.20), and biochanin A (log_2_FC = 2.19). Hydroxycinnamic acid conjugates, including coumaroyl and sinapoyl aspartate derivatives (log_2_FC = 2.76–5.09) and sinapic acid (log_2_FC = 3.70), were additionally enriched.

In contrast, epicotyl-derived calli were characterized by a higher content in coumaroyl and feruloyl hexoside conjugates, including feruloyl dihexoside (log_2_FC = −7.47) and multiple coumaroyl-hex-coumaroyl glycoside variants (log_2_FC = −1.59 to −4.98). Several genistein and apigenin derivatives were also higher in epicotyl-derived calli, including 2′-hydroxygenistein malonylhex-[hex coumaric acid] (log_2_FC = −1.94, *p* = 1.11 × 10^−6^) and apigenin malonylhex-CO_2_ (log_2_FC = −1.54).

Direct comparison between the two cotyledon-derived lines (Bolero 4-1C and Bolero 1-1C) revealed marked reciprocal shifts across multiple specialized metabolite classes. Bolero 4-1C had a higher content in lignans and hydroxycinnamic acid derivatives, most prominently syringaresinol isomers (log_2_FC up to 22.18) and syringaresinol hexoside (log_2_FC = 7.29), alongside elevated levels of caffeic acid, ferulic acid, sinapic acid isomers, and sinapoylglucose conjugates (log_2_FC = 3.2–9.3). Several isoflavonoids, including formononetin and daidzein isomers, as well as lupinisoflavone malonylhexosides, were also significantly increased (log_2_FC = 4.0–9.6). In contrast, Bolero 1-1C was characterized by a higher abundance of saponins, including soyasapogenol and bayogenin derivatives (log_2_FC down to −17.06), together with medicagenic acid derivatives. Additional lower contents in 4-1C relative to 1-1C were observed for feruloyl dihexoside (log_2_FC = −8.37), malonyl ethyl dihexoside (log_2_FC = −6.17), and phospholipids such as PG (18:2/0:0) and PI (18:3/0:0) isomers (log_2_FC ≈ −14.6 to −16.6). Collectively, these results demonstrate a clear metabolic contrast between the two cotyledon-derived lines, with one preferentially richer in hydroxycinnamic acids and lignans and the other richer in saponins.

### 2.7. Integration of Metabolomic Data with Biomass and Antioxidant Activity (Ferric Reducing Capacity)

To investigate which of the identified metabolites contribute to the measured ferric reducing capacities of the extracts, Spearman correlation analysis was conducted between FRAP values and the annotated metabolite matrix across all samples (*n* = 30), with Benjamini–Hochberg correction applied to control the false discovery rate. Ferric reducing capacity exhibited extensive covariation with metabolomic composition. Of the 290 metabolites tested, 196 were significantly associated with the measured FRAP values, indicating the general coupling between redox capacity and metabolite content. Using effect-size thresholds, 120 metabolites displayed |r| ≥ 0.6, including 82 with |r| ≥ 0.7 and 40 with |r| ≥ 0.8. Metabolites positively associated with FRAP are phenylpropanoid-derived compounds, including isoflavonoids (e.g., daidzein and biochanin A derivatives, genistein glycosides), prenylated flavonoids, and hydroxycinnamic acids, as well as some phospholipids and oxidized fatty acids. In contrast, negative correlations were predominantly for malonylated or glycosylated flavonoid conjugates and saponins, suggesting structural subclass specificity in redox contribution rather than uniform behaviour across secondary metabolites.

Because FRAP values differed among species, pathway-level analyses were performed using relative class abundances. For each sample, metabolite intensities belonging to the same biochemical class were summed and normalized to the total annotated metabolite intensity, generating proportional contributions from each class to the overall metabolomic profile. These relative proportions were then correlated with FRAP values to assess whether ferric reducing capacity was associated with shifts in pathway-level allocation rather than individual metabolite abundance. Across all samples (*n* = 30), FRAP exhibited significant negative correlations with the relative abundance of saponins (r = −0.53, *p* = 0.003), total lipids (r = −0.41, *p* = 0.026), and primary metabolites/redox pool (r = −0.39, *p* = 0.031). While FRAP was positively correlated with the relative abundance of alkyl glycosides (r = 0.44, *p* = 0.015). No significant correlations were detected for total isoflavonoids, total flavonoids, or total hydroxycinnamic acid derivatives at the pathway level. These findings indicate that interspecific variation in ferric reducing capacity is structured by differences in relative metabolic abundance among major biochemical branches rather than by uniform scaling of total flavonoid or phenylpropanoid abundance. To distinguish interspecific from intraspecific effects, correlation analysis was repeated within soybean samples only. After FDR correction, only one metabolite remained significantly associated with FRAP, and no major metabolite class showed significant pathway-level associations.

## 3. Discussion

Callus induction is a hormone-driven dedifferentiation process in which auxin–cytokinin signalling reprograms somatic cells into a dedifferentiated proliferative state, although hormonal requirements and tissue responsiveness vary considerably among species, genotypes, and explant types [24]. While specialized metabolite production in plant callus cultures has been extensively documented [6,24,25], whether lineage-specific biosynthetic identities are preserved or reprogrammed during dedifferentiation, and how intraspecific factors further shape metabolic output, remains insufficiently understood. Previous in vitro metabolomic studies in legumes focused on soybean, where isoflavonoids are consistently reported as the principal specialized metabolites of callus and suspension cultures [26,27]. For lupin and pea, metabolomic characterization of calli remains limited, with most studies addressing induction efficiency, regeneration capacity, or physiological responses rather than comprehensive metabolite profiling [26,28]. Analyses of differentiated *Lupinus* tissues consistently identify saponins and isoflavones as dominant metabolite classes [18,29,30], whereas vegetative tissues of *P. sativum* are characterized by high abundances of hydroxycinnamates, including p-coumaric, ferulic, and sinapic acid derivatives [31]. Although a recent multi-omics study has begun to address species-specific metabolic differences in callus cultures across phylogenetically distant species [32], whether lineage-specific biochemical tendencies are preserved during dedifferentiation within a single plant family, and how genotype, explant origin, and independent line establishment modulate such profiles, has not previously been examined under standardized conditions in Fabaceae.

In the present study, callus cultures from three phylogenetically distinct and agronomically important Fabaceae species, *G. max*, *L. angustifolius*, and *P. sativum*, established under identical hormonal conditions, were subjected to untargeted UHPLC–MS/MS metabolomic profiling, enabling a direct comparison of secondary metabolite profiles across multiple legume species in dedifferentiated cells. Our results demonstrate that species identity is the primary determinant of the metabolic profile of legume callus cultures: soybean calli were characterized by saponin dominance with the accumulation of soyasapogenol-saponins, lupin calli by an isoflavonoid and saponin specialization, with lignan accumulation observed specifically in cotyledon-derived lines, and pea calli by a hydroxycinnamate-centred profile. These findings indicate that species-specific biosynthetic tendencies are largely retained in dedifferentiated tissues under standardized hormonal conditions, rather than fully reset by hormonal reprogramming. These results establish a metabolomic baseline for the use of legume callus cultures as model systems for studying species-specific specialized metabolism in dedifferentiated tissues, providing a foundation for future biotechnological development.

Callus induction efficiency and subsequent morphophysiological development differed substantially among species, genotypes, and explant types despite identical hormonal treatment. Complete induction was achieved in cotyledon explants of Bolero and Karacter, whereas Nessie cotyledons induced calli in approximately 50% of explants and no callogenesis was observed for Tofina cotyledon explants. The complete recalcitrance of Tofina cotyledons under conditions effective in other genotypes indicates that tissue-intrinsic factors in certain genotypic backgrounds can override exogenous hormonal induction [33].

Beyond induction efficiency, callus morphology diverged markedly among species. Soybean calli remained white and friable throughout culture, whereas lupin calli developed brown pigmentation once growth was established, and pea calli acquired a reddish-brown compact nodular morphology already during early proliferation. Untargeted metabolomic profiling supports a mechanistic interpretation of these contrasting phenotypes. Lupin calli are rich in isoflavonoids and pea in hydroxycinnamic acid derivatives, which serve as substrates for peroxidase- and polyphenol oxidase-mediated oxidative polymerisation [34,35]. In lupin, browning appeared only after substantial biomass accumulation, consistent with the view that oxidative phenolic metabolism followed—rather than constrained—the proliferative phase, as reflected by the relatively high fresh weights recorded in Bolero calli. In pea, by contrast, pigmentation coincided with the proliferative phase itself and with the lowest biomass recorded across all lines (Table 1), indicating that early phenylpropanoid-driven oxidative activity may have limited cell division. A comparable inverse relationship between hydroxycinnamate accumulation and mitotic activity has been reported in embryogenic suspensions of alfalfa (*Medicago sativa* L.) [36].

Untargeted metabolomic profiling revealed that the main determinant for the callus metabolome is the species from which the calli were initiated. Principal component analysis of the 290 curated metabolites showed that species identity was the dominant structuring factor of the metabolome space, with PC1 and PC2 together explaining 71.9% of the variance and samples clustering tightly according to species (Supplemental Figure S3). This separation reflects coordinated differences in the activity of entire biosynthetic pathways rather than variations in individual metabolites. Soybean calli were dominated by glycosylated soyasapogenol B and E derivatives, consistent with their reported abundance in soybean tissues [16,17]. In contrast, lupin calli were characterized by flavonoids and isoflavones, including lupinalbin A, lupinisoflavone B and D glycosides, and luteone derivatives—which are a prenylated isoflavonoid characteristic of *Lupinus* species with documented antifungal and antioxidant activity [37]. In addition to flavonoids, saponins derived from soyasapogenol B and hederagenin, as previously described in lupin tissues, were also tentatively identified [18,35]. Pea calli, by comparison, were rich in hydroxycinnamate conjugates and specific flavonoids—including coumaroyl methylhexose derivatives—and distinct tricin and biochanin A isomers, in agreement with metabolite profiles reported in pea seeds and cotyledons [20,31,38]. Despite these species-specific profiles, shared metabolic features were also observed. Both soybean and blue lupin accumulated soyasapogenol B derivatives as dominant metabolites, differing primarily in their glycosylation patterns, indicating that interspecific variation arises mainly from terminal glycosylation rather than the early steps of the triterpene biosynthetic pathway. Similarly, a soyasapogenol E–derived saponin was identified as a dominant metabolite in both pea and soybean, although present as different isomers in each species. This suggests that while the core structure and glycosylation composition are conserved, species-specific differences result from variation in glycosidic linkage positions or stereochemistry. The persistence of these species-associated metabolic signatures in undifferentiated tissues suggests that dedifferentiation reorganizes transcriptional programmes linked to cell proliferation while preserving elements of lineage-specific biosynthetic regulation. This observation is consistent with evidence that chromatin remodelling during callogenesis is selective rather than global [22].

The enrichment of saponins in soybean calli, particularly in epicotyl-derived lines, represents one of the most distinctive metabolic features observed in this study. Previous in vitro investigations of soybean have largely emphasized isoflavonoids such as daidzein, genistein, and their glycosidic and malonylated derivatives, as the principal specialized metabolites of callus and suspension cultures [39,40], whereas triterpenoid saponins have received comparatively little attention in soybean in vitro systems. In the present dataset, glycosylated soyasapogenol B and E derivatives were among the strongest discriminant metabolites in interspecific comparisons (F > 400; FDR < 10^−18^), indicating that the β-amyrin-derived triterpenoid pathway remains active in dedifferentiated soybean tissue. Epicotyl-derived calli of Nessie were particularly enriched in soyasapogenol derivatives relative to cotyledon-derived calli, consistent with the known tissue-dependent distribution of soyasaponins in intact plants, where vegetative organs often exhibit elevated saponin profiles relative to seeds. Conversely, acetylated group A saponins, characteristic constituents of soybean hypocotyl and cotyledon, were not detected in any callus line, indicating that terminal acetylation steps are not maintained in undifferentiated cells, consistent with their reported developmental regulation in intact plants [41,42].

While species identity structured the global metabolic profile, intraspecific variance was superimposed on this framework. In soybean, the abundance of 109 metabolites differed significantly between Nessie and Tofina, with Nessie calli enriched in isoflavonoids (formononetin) and soyasapogenol conjugates, whereas Tofina accumulated higher levels of free and conjugated hydroxycinnamate derivatives, including sinapic acid and feruloyl dihexoside. Explant origin further contributed to metabolic divergence: epicotyl-derived Nessie calli differed from cotyledon-derived tissues by 56 metabolites and showed coordinated enrichment of saponins and hydroxycinnamate conjugates [41]. In *L. angustifolius*, divergence among independently established callus lines from the same donor plant was even more pronounced. Bolero 1-4C was enriched in lignans and hydroxycinnamic derivatives, whereas Bolero 1-1C exhibited saponin dominance, including soyasapogenol and bayogenin derivatives. Such line-level divergence is consistent with somaclonal epigenetic variation arising during in vitro culture [43,44]. Nevertheless, the persistence of clear species-level clustering in multivariate space despite this within-species variability indicates that lineage-associated metabolic organization remains robust.

The integration of metabolomic data with ferric reducing capacity measurements revealed that FRAP values were determined by the structural identity of dominant metabolite subclasses rather than by overall phenolic accumulation. Although the abundance of numerous individual annotated metabolites showed significant correlation with FRAP values, no significant correlations were detected for total isoflavonoids, total flavonoids, or total hydroxycinnamic acids as metabolite classes. Instead, significant negative pathway-level correlations were observed for saponins and lipids. At the individual metabolite level, positive associations with FRAP were observed for specific phenylpropanoid-derived compounds, including free or lightly conjugated isoflavonoids, prenylated flavonoids, and hydroxycinnamic acids [18,45], while malonylated or highly glycosylated flavonoid conjugates and saponins showed negative correlations. This subclass-level pattern is consistent with established structure–activity relationships in which free phenolic hydroxyl groups are the primary determinants of radical-scavenging capacity [46,47]; glycosylation at key positions has been shown to attenuate this activity by masking reactive sites [48]. The negative correlation with saponins and lipids likely reflects a compositional dilution effect: lines enriched in these FRAP-inactive compound classes contain proportionally less redox-active phenolic compounds. Importantly, when the correlation analysis was restricted to within-soybean comparisons, most FRAP–metabolite correlations disappeared, indicating that the correlations identified across the full dataset are predominantly driven by interspecific differences rather than by direct, mechanistic relationships between individual metabolites and reducing capacity at the within-species level. The FRAP–metabolite associations reported here should therefore be interpreted as reflecting species-level metabolic orientation (specifically the relative allocation between polyphenolic and triterpenoid biosynthetic pathways) rather than as a quantitative, within-species relationship between specific compounds and ferric reducing capacity.

Several limitations of the present study must be considered. First, metabolomic profiles were characterized exclusively in callus cultures, without direct comparison to differentiated tissues of the same genotypes. It is known that dedifferentiation alters the regulatory constraints governing secondary metabolism, and that callus cultures may exhibit quantitative and qualitative shifts relative to the organs from which they were derived [49]. Callus cultures, being in an undifferentiated state, may lack the regulatory constraints and conserved signalling networks observed in differentiated tissues, potentially resulting in greater molecular differences between species [32]. Future comparative studies, including differentiated tissues, will therefore be essential to fully resolve the extent to which the lineage-specific metabolic signatures observed here are conserved relative to the intact plant. Second, the biological design of this study was inherently unbalanced, with two cultivars and multiple lines for soybean, one cultivar and multiple lines for lupin, and only one line for pea. The calli described in this study are those calli resulting from trails on a broader range of Fabaceae (Supplemental Table S1). Using the hormonal conditions described here, no proliferation response was observed in *Vicia faba* or *Cicer arietinum*, while few could be generated from *P. sativum*. Optimization of the hormonal treatment or of the callogenesis conditions in general will potentially enlarge the genetic diversity of the studied calli, but direct comparison of calli generated using different hormone treatments is not possible. As a consequence of having only one line of pea, conclusions for pea must be handled with care and generalization avoided; nonetheless, the distinct hydroxycinnamate-centred profile observed in this line is consistent with phenylpropanoid-dominant metabolism reported in differentiated pea tissues. Finally, the callus lines analyzed had been maintained over approximately two years (~16–20 successive subcultures), and the metabolomes reported here only reflect those of long-term stabilized cultures rather than the immediate post-induction metabolic state. Prolonged in vitro maintenance is known to promote somaclonal variation and epigenetic drift [44], processes that are consistent with the line-to-line variability observed here among independently established lines derived from the same donor plant and explant type. Together, these considerations delimit the scope of our conclusions while highlighting concrete avenues for future work, including the inclusion of source-tissue comparisons, broader cultivar sampling, and time-resolved metabolomic monitoring across passages.

## 4. Materials and Methods

### 4.1. Plant Material and Culture Conditions

Five Fabaceae species were used for the establishment of in vitro cultures: soybean, represented by two cultivars (“Nessie” and “Tofina”); blue lupin cultivar “Bolero”; the pea cultivars “Karacter” and Greenway; the faba bean cultivars “Magnolia” and “Jasmin”; and the chickpea cultivars Elmo and Orion (Supplemental Table S1). Seeds were provided by the Institute for Organic Agriculture and Agroecology Luxembourg a.s.b.l. (IBLA). Prior to culture initiation, seeds were surface sterilized under aseptic conditions following the method described by Dodds and Roberts (1985) [50] with minor modifications, consisting of immersion in 70% (*v*/*v*) ethanol for 1 min, followed by treatment with 0.8% (*w*/*v*) sodium hypochlorite for 20 min, and five rinses with sterile distilled water to remove residual disinfectant. Sterilized seeds were placed on solid Murashige and Skoog (MS) basal medium supplemented with 30 g L^−1^ sucrose and solidified with 7 g L^−1^ agar. The pH of the medium was adjusted to 5.8 ± 0.1 using 0.5 M KOH prior to autoclaving at 121 °C for 20 min. Cultures were maintained in a controlled growth chamber at 27 ± 2 °C under a 16 h light/8 h dark photoperiod.

### 4.2. Callus Induction

Well-developed seedlings exhibiting fully expanded cotyledons were used as the source of explants. Cotyledons were excised and longitudinally sectioned along the midrib into approximately 1 cm segments. Each explant was placed with the abaxial surface in contact with full-strength MS medium supplemented with vitamins, 2,4-dichlorophenoxyacetic acid (2,4-D; 1 mg L^−1^), and kinetin (0.2 mg L^−1^) [51]. We followed the general callus induction approach described by Dodds and Roberts [50]. The medium contained 30 g L^−1^ sucrose and 7 g L^−1^ agar, with the pH adjusted to 5.8 using 1 N KOH. Media were sterilized by autoclaving at 121 °C for 20 min. Three to five explants were placed per Petri dish and incubated in the dark at room temperature.

After excision of the cotyledons, the remaining seedlings were maintained on MS medium (30 g L^−1^ sucrose, 7 g L^−1^ agar) under the same growth conditions to support continued development. After 15 days, epicotyl segments were excised, cut into 1 cm fragments, and cultured vertically on MS medium supplemented with 2,4-D (1 mg L^−1^) and kinetin (0.2 mg L^−1^). Three to five epicotyl explants were placed per Petri dish and incubated in the dark at room temperature.

### 4.3. Callus Maintenance and Biomass Measurement

After 60 days, callus formation was evaluated based on induction frequency, colour, and consistency (Supplemental Table S1). Successfully induced calli were carefully excised from the original explants and subcultured as independent tissues onto fresh MS medium with the same hormone composition, following standard callus maintenance procedures [51]. All callus lines that proliferated and were selected for metabolomic analyses were established at the same time and maintained for approximately two years under identical conditions, corresponding to approximately 16–20 successive passages. Samples were collected at the end of a 5–6-week subculture cycle, immediately prior to the next scheduled subculture, ensuring that all lines were harvested at a comparable developmental and metabolic state. For metabolomic analysis, proliferating calli were selected from all trials initiated. Those calli that proliferated allowed us to perform systematic comparisons across multiple biological levels: species, cultivars within the same species, explant origins within the same cultivar, and independent lines derived from the same plant and explant type. This hierarchical selection scheme was designed to capture the full range of metabolic variation attributable to taxonomic, genotypic, and ontogenetic factors. For each selected callus line, fresh weight was first recorded; samples were then lyophilized to constant weight, and the dry callus was measured prior to downstream untargeted metabolomic profiling [25].

### 4.4. Metabolite Extraction

Callus samples were lyophilized using a Martin Christ freeze-dryer (Christ Alpha 2-4 LCS Plus, Osterode am Harz, Germany). Lyophilized calli were ground into a fine powder using metallic beads. An amount of 50 mg of the powdered material was weighed for extraction. Metabolites were extracted with 80% methanol (MeOH) and 20% Milli-Q water (Merck Millipore, Darmstadt, Germany) at a solvent-to-sample ratio of 12:1 (*v*/*w*). The mixture was agitated on a Thermomixer (Eppendorf, Hamburg, Germany) at 25 °C for 4 h. Following extraction, samples were centrifuged at 14,000 rpm for 25 min at 4 °C. Supernatants were collected and stored at −20 °C until further untargeted metabolomic analysis [18].

### 4.5. Untargeted Metabolomics Analysis with UHPLC-MS/MS and Identification

The extracts, initially extracted in 80% MeOH, were diluted with Milli-Q water to attain a final concentration of 20% MeOH/MQ. A quality control (QC) sample was composed of 10 μL of each of the extracts, this sample was run before the batch and repeatedly every sixth sample during the sequence to monitor the instrumental stability and analytical reproducibility. The diluted extracts were filtered through a 0.22 μm PTFE syringe filter (Millex-LG, Merck KGaA, Darmstadt, Germany) and analyzed using an Acquity UPLC I-Class system equipped with a diode array detector (Waters, Milford, MA, USA) coupled to a 6600+ TripleTOF hybrid quadrupole-time of flight mass spectrometer (SCIEX, Framingham, MA, USA) in the positive and negative ionization modes [52]. Ten microliters of the sample were separated on a reverse-phase Acquity UPLC BEH C18 column (2.1 × 100 mm, 1.7 μm particle size) (Waters, Milford, MA, USA) with a column temperature of 50 °C and a flow rate of 0.5 mL/min. The mobile phase consisted of 0.1% (*v*/*v*) formic acid in water (A) and 0.1% (*v*/*v*) formic acid in acetonitrile (B), with the following gradient: 0 min, 1% B; 4 min, 1% B; 16 min, 5% B; 35 min, 40% B; 45 min, 100% B; 50 min, 100% B; 54 min, 1% B; and 60 min, 1% B. UV–visible spectra were acquired between 190 and 800 nm at a rate of 10 points/s.

Electrospray ionization was performed using the following parameter values for the positive and negative modes: source temperature 650 °C; ion spray voltage of 4.5 and −4.5 kV, respectively; curtain gas (nitrogen) of 30 psi; nebulizer gas (air) of 55 psi; and turbine gas (air) of 50 psi. The declustering potential was set up at 60 V in the positive and −60 V in the negative mode. Survey scans of 175 ms were acquired for information-dependent acquisition. The ten highest MS ions were selected for fragmentation if they were singly charged and had an intensity exceeding a threshold of 100 counts/s. Product ion scans were collected with an accumulation time of 200 ms in high-sensitivity mode, thus leading to a total cycle time of 2.225 s. A sweeping collision energy of 15 V below and above 15 V in the positive and −15 V in the negative mode was applied to all precursor ions. The dynamic exclusion was set for 2 s after three occurrences before the precursor could be fragmented again. Data for all cultivars were acquired in the negative mode, while positive mode analysis was run on one cultivar of each species to aid in the identification of the compounds. The correlation between the QC samples was calculated to be above 98% over the batch, with below 75% correlation calculated between the QC samples and the other samples.

For the identification and relative quantification, raw data files were uploaded in Progenesis QI (v2.3, Nonlinear Dynamics, Newcastle upon Tyne, UK) to align all runs, normalize the data, and perform relative quantitative analysis based on sample groups (species). Only features with MS/MS data were retained for the identification stage. The output data were manually reviewed using PeakView software (v1.2, SCIEX, Framingham, MA, USA). Initial identification relied on the use of an in-house database exported in msp-format containing mainly saponins previously identified in seven Fabaceae species, including soybean, lupin (white and blue species) and pea. All database hits were manually validated. For compounds not present in this in-house database, identification was achieved through a literature search, and MS/MS comparison using databases such as GNPS (https://gnps.ucsd.edu/ProteoSAFe/libraries.jsp), MZCloud™ (https://beta.mzcloud.org/), LipidMaps (https://www.lipidmaps.org/), and PubChem (https://pubchem.ncbi.nlm.nih.gov). Accepted identifications were subsequently incorporated into the in-house database for future dereplication. All identifications reported in this study adhere to level 2 standards as defined by the Metabolomics Standards Initiative (MSI). Following tentative annotation, redundant features corresponding mainly to dimers and adducts were identified during compound-by-compound MS/MS-based manual curation and removed, yielding the final non-redundant curated dataset used for all downstream multivariate analyses. A detailed description of the data-processing workflow is provided in Supplementary Note S1. Representative total ion chromatograms (TICs) in the negative ESI mode for one sample per species are provided in Supplementary Figure S1.

### 4.6. Ferric Reducing/Antioxidant Power Assay

The FRAP assay, which measures antioxidant capacity based on the reducing power of molecules to convert Fe(III) to Fe(II) [53], was selected as it specifically captures electron transfer-based reducing power, which is the predominant antioxidant mechanism of the isoflavonoids, hydroxycinnamic acids, and flavonoids identified in the metabolomic profiles of our Fabaceae calli, and has been widely validated for legume-derived extracts, allowing direct comparison with published values [20,54]. For the assay, 10 μL of the extracted samples were mixed with 190 μL of freshly prepared FRAP reagent (300 mM acetate buffer, 10 mM ferric-tripyridyltriazine, 20 mM FeCl_3_; pH 3.6) in a 96-well plate. The plate was covered with aluminum foil and incubated at room temperature for 20 min. A standard curve was prepared using FeSO_4_ at varying concentrations (0, 0.05, 0.1, 0.2, 0.4, 0.6, 0.8, and 1 mM), yielding the equation y = 0.6787x + 0.077 (R^2^ = 0.9981). Ferric reducing capacity was expressed as µM ferrous equivalents. Absorbance was measured at 595 nm using a spectrophotometric microplate reader (Spark, Tecan, Männedorf, Switzerland).

### 4.7. Statistical Analysis

The data were analyzed to assess differences between the different callus lines and within each cultivar–species. Statistical analysis of metabolite content was initially performed in Progenesis QI, where features with a *p*-value < 0.05 were selected using an internally applied one-way ANOVA. To further explore variation across the samples, multivariate and univariate analyses were performed using MetaboAnalyst 6.0 (www.metaboanalyst.ca). The data were normalized by median and subjected to square root transformation followed by Pareto scaling to improve data comparability. All multivariate and univariate analyses were performed in MetaboAnalyst 6.0. Prior to analysis, data were log-transformed and Pareto-scaled. Principal component analysis (PCA) and hierarchical clustering analysis (HCA) were used as unsupervised methods to explore the global structure of the dataset and to evaluate the relative contribution of specialized versus primary metabolism to species-level grouping. Partial least squares discriminant analysis (PLS-DA) was performed and evaluated by leave-one-out cross-validation, reporting the cumulative goodness-of-fit (R^2^), the cross-validated predictive ability (Q^2^), and classification accuracy. Permutation testing was conducted with 1000 permutations using prediction accuracy as the test statistic. Variable importance in projection (VIP) scores were computed for feature ranking. For univariate analyses, *p*-values were adjusted for multiple testing using the false discovery rate (FDR) correction according to the Benjamini–Hochberg procedure. Correlation analysis was conducted using the Spearman rank correlation test on normalized metabolite data to evaluate the relationship between metabolite abundance and ferric reducing capacity using OriginPro 2024. Ferric reducing capacity was analyzed separately using one-way ANOVA followed by Tukey’s post hoc test (*p* < 0.05) in OriginPro 2024.

## 5. Conclusions

This study provides the first comparative metabolomic characterization of callus cultures from three Fabaceae species maintained under uniform culture conditions, demonstrating that species-specific metabolic signatures are retained in dedifferentiated tissues. Despite hormonal reprogramming, each species retained a distinct chemical signature. The metabolomes of soybean calli were dominated by saponins, lupin calli by isoflavonoids and saponins accompanied by lignan accumulation, and pea calli were characterized by a hydroxycinnamate-centred phenylpropanoid metabolome. Within this species-defined metabolic profile, additional quantitative variation arose from genotype, explant origin, and independent line establishment. Ferric reducing capacity differed substantially but not significantly across species and was associated with the structural identity of dominant metabolite subclasses rather than overall metabolite abundance; this association was largely driven by interspecific differences rather than by direct within-species relationships. Importantly, these findings underscore the necessity of preserving and characterizing individual callus lines, as metabolite profiles were not necessarily reproducible even among lines derived from the same donor plant and explant type. Together, these results establish a metabolomic baseline for three agronomically important legume species and highlight the value of Fabaceae callus cultures as tractable model systems for studying species-specific specialized metabolism in dedifferentiated tissues. Future integration of transcriptomic and epigenomic analyses will be essential to resolving the regulatory mechanisms underlying these metabolic patterns, to identifying biosynthetic control points amenable to rational metabolic engineering, and to better understanding the sources of line-to-line metabolic instability during in vitro culture.

## Figures and Tables

**Table 1:** Callus induction efficiency, fresh and dry biomass yield, and morphological characteristics of calli derived from cotyledon and epicotyl explants in three Fabaceae species.

Species	Calli Line	Explant	Induced Calli (%)	Mean Fresh Weight (g), SD	Mean Dry Weight (g), SD	Callus Characteristics
Soybean (*G. max*)	Nessie 2.1CA	Cotyledon	50%	2.7 ± 0.13 ^a^	0.17 ± 0.01 ^ab^	White, soft, and friable
	Nessie 2.1CB	Cotyledon	50%	1.82 ± 0.25 ^b^	0.15 ± 0.01 ^abc^	White, soft, and friable
	Nessie 4.1 C	Cotyledon	50%	2.38 ± 0.13 ^b^	0.19 ± 0.03 ^a^	White, soft, and friable
	Nessie 4.1T	Stem	100%	2.26 ± 0.34 ^b^	0.14 ± 0.02 ^abc^	White, soft, and friable
	Tofina C	Cotyledon	0%	-	-	-
	Tofina 1.3T	epicotyl	100%	3.01 ± 0.07 ^a^	0.21 ± 0.01 ^a^	White, soft, and friable
	Tofina 1.2T	epicotyl	100%	2.62 ± 0.51 ^a^	0.17 ± 0.02 ^ab^	White, soft, and friable
Blue lupin (*L. angustifolius*)	Bolero 1.4C	Cotyledon	100%	4.93 ± 2.37 ^a^	0.13 ± 0.08 ^abc^	White turns to brownish; soft, and friable
	Bolero 1.1C	Cotyledon	100%	2.26 ± 0.46 ^b^	0.16 ± 0.03 ^abc^	White turns to brownish; soft, and friable
	Bolero 1.2 T	epicotyl	70%	1.11 ± 0.39 ^b^	0.07 ± 0.03 ^c^	White turn to brownish; soft, and friable
Pea (*P. sativum*)	Karacter 6.1C	Cotyledon	100%	-	-	Brownish, no proliferation
	Karacter 3.2T	epicotyl	84%	1.13 ± 0.33 ^b^	0.09 ± 0.01 ^bc^	Yellowish turn to reddish/brownish; compact, and nodular

Callus line codes are structured as follows: [Cultivar name] [Donor plant number] [Explant number] [Explant type] [Callus letter]. Explant type: C = cotyledon, T = epicotyl. When two callus masses developed independently on the same explant piece, they are distinguished by a trailing letter (A or B). For example, Nessie 4.1CA and Nessie 4.1CB designate two independent callus masses derived from explant 1 of donor plant 4 of the Nessie cultivar (cotyledon origin). “-” indicates that callus induction was unsuccessful or that induced calli failed to establish after transfer to solid medium. Specifically, Tofina cotyledon explants yielded a callus induction rate of 0%, while Karacter cotyledon-derived calli—although initially induced—failed to proliferate upon subculture, becoming brownish and compact, and were therefore excluded from all subsequent biomass, metabolomic, and antioxidant analyses. Statistical differences among lines were determined using one-way ANOVA followed by Tukey’s post hoc test (*p* ≤ 0.05) (*n* = 3), statistical differences between the calli are indicated by superscript letters.

## Data Availability

The original contributions presented in this study are included in the article/Supplementary Materials. Further inquiries, including to obtain the raw datasets at the base of the current study, can be directed to the corresponding author.

## Supplementary Materials

The following supporting information can be downloaded at: https://www.mdpi.com/article/10.3390/plants15152299/s1, Figure S1: Representative total ion chromatograms (TICs) of callus extracts from one sample per species in negative ESI mode; Figure S2: Representative images of the callus cultures derived from different Fabaceae species; Figure S3: Principal component analysis (PCA) of the 290 curated metabolites; Figure S4: Hierarchical clustering analysis (HCA) of the 290 curated metabolites; Figure S5: PCA of callus samples based on the specialized metabolite subset (*n* = 234); Figure S6: HCA of callus samples based on the specialized metabolite subset (*n* = 234); Figure S7: PCA of callus samples restricted to the saponin subset (*n* = 38); Figure S8: HCA of callus samples restricted to the saponin subset (*n* = 38); Figure S9: PCA of callus samples restricted to the flavonoid and isoflavonoid subsets; Figure S10: HCA of callus samples restricted to the flavonoid and isoflavonoid subsets; Figure S11: PCA of callus samples restricted to the hydroxycinnamic acid subset (*n* = 58); Figure S12: HCA of callus samples restricted to the hydroxycinnamic acid subset (*n* = 58); Figure S13: PCA of callus samples restricted to the primary metabolite subset (*n* = 56); Figure S14: HCA of callus samples restricted to the primary metabolite subset (*n* = 56); Figure S15: PLS-DA cross-validation performance plot; Figure S16: PLS-DA permutation test (1000 permutations); Table S1: RSD values of detected features in QC samples; Table S2: Complete annotated metabolite dataset; Supplementary Note S1: Untargeted metabolomic data-processing and filtering workflow.

## Abbreviations

The following abbreviations are used in this manuscript:

*G. max*	*Glycine max* (soybean)
*L. angustifolius*	*Lupinus angustifolius* (blue lupin)
*P. sativum*	*Pisum sativum* (pea)
UHPLC–MS/MS	Ultra-High Performance Liquid Chromatography–tandem Mass Spectrometry
ESI	Electrospray Ionization
PCA	Principal Component Analysis
PLS-DA	Partial Least Squares Discriminant Analysis
HCA	Hierarchical Clustering Analysis
FRAP	Ferric Reducing/Antioxidant Power
VIP	Variable Importance in Projection
FDR	False Discovery Rate
ANOVA	Analysis of Variance
RDA	Retro-Diels–Alder
QC	Quality Control
UV	Ultraviolet
RT	Retention Time
PC1/PC2	Principal Component 1/Principal Component 2
SD	Standard Deviation
FC	Fold Change
MSI	Metabolomics Standards Initiative
MeOH	Methanol
MS	Murashige and Skoog (medium)
2,4-D	2,4-Dichlorophenoxyacetic acid
Hex	Hexose
dHex	Deoxyhexose
Pent	Pentose
HexA	Hexuronic acid
HMG	Hydroxymethylglutaryl
FeSO_4_	Ferrous sulfate
KOH	Potassium hydroxide
PTFE	Polytetrafluoroethylene
IBLA	Institute for Organic Agriculture and Agroecology Luxembourg a.s.b.l.
GNPS	Global Natural Products Social Molecular Networking
