# Supplementary material for: Species-Specific Metabolic Identities Persist in Fabaceae Callus Cultures Under Standardized Culture Conditions
Source: Plants (Basel). 2026 Jul 27;15(15):2299. doi: 10.3390/plants15152299 (PMC13468598; doi:10.3390/plants15152299)
Supplement: Supplementary file 1 [file plants-15-02299-s001.zip › Supplementary (figures) revision.pdf]

Supplementary material:

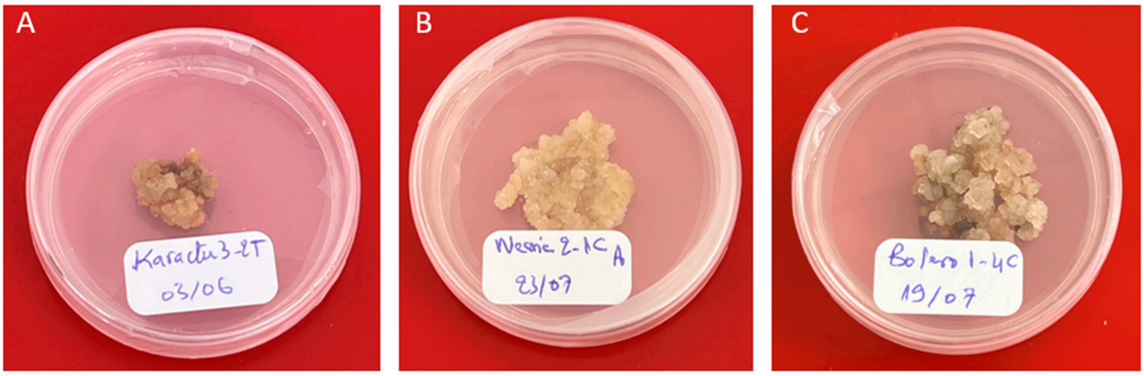

Figure S1. Representative callus cultures derived from different Fabaceae species. (A) *P. sativum* cv. Karacter; (B) *G. max* cv. Nessie; (C) *L. angustifolius* cv. Bolero. Calli were cultured in MS medium supplemented with 2,4-D (1 mg L^{-1}) and kinetin (0.2 mg L^{-1}) and photographed after three weeks of growth, showing species-dependent differences in morphology, texture, and pigmentation.

(A)

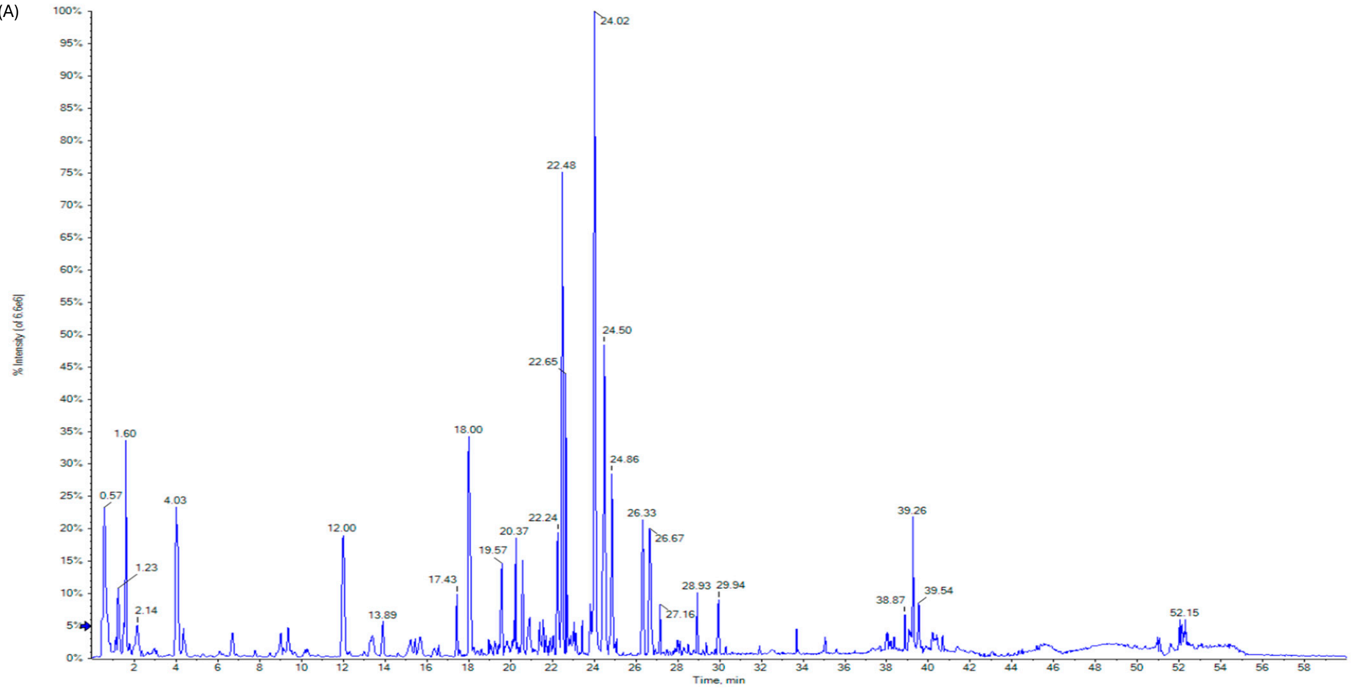

% Intensity (of 6.6e6)
Time, min
24.02
22.48
24.50
22.65
1.60
18.00
24.86
0.57
4.03
26.33
39.26
12.00
22.24
20.37
26.67
19.57
1.23
17.43
28.93
29.94
39.54
38.87
2.14
13.89
27.16
52.15

(B)

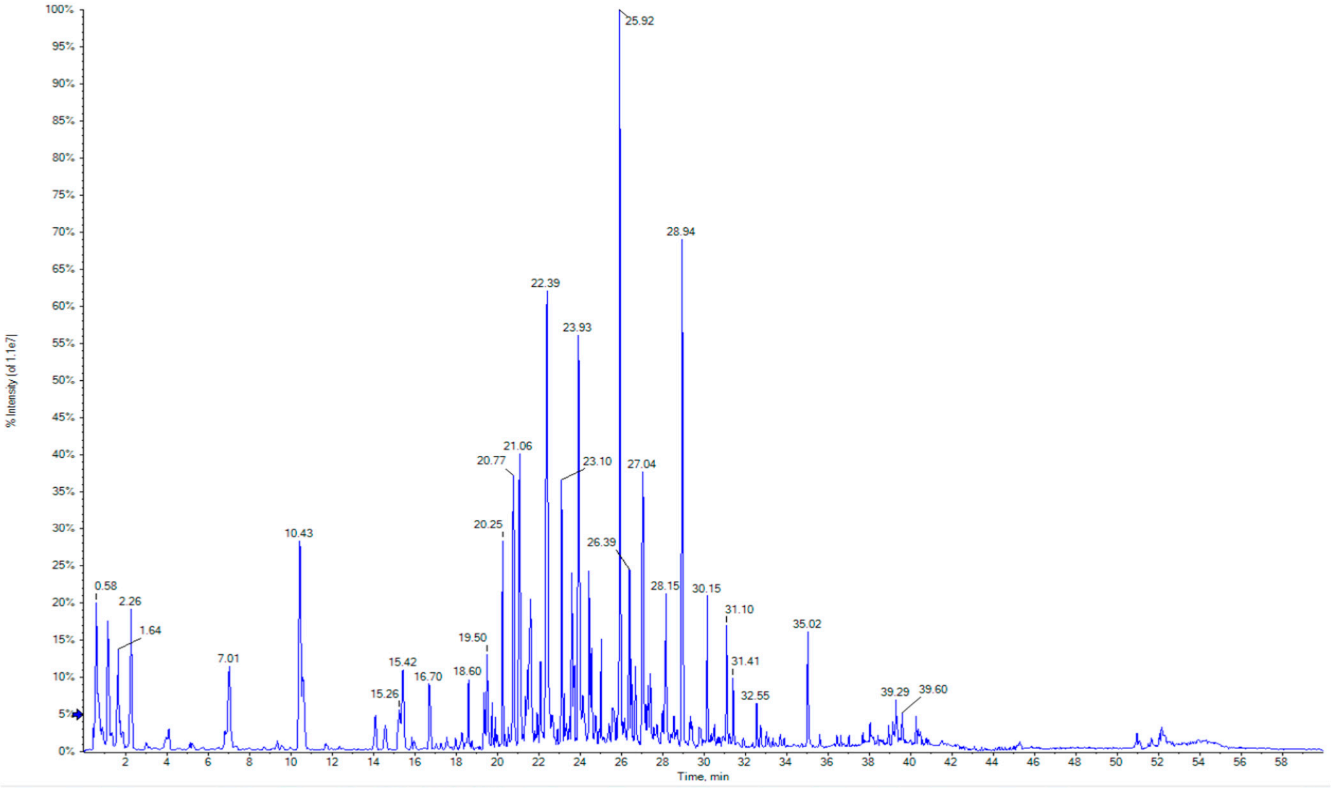

% Intensity (of 1.1e7)
Time, min
25.92
28.94
22.39
23.93
21.06
20.77
23.10
27.04
20.25
10.43
26.39
0.58
2.26
28.15
30.15
1.64
31.10
35.02
7.01
19.50
15.42
31.41
16.70
18.60
39.29
39.60
15.26
32.55

(C)

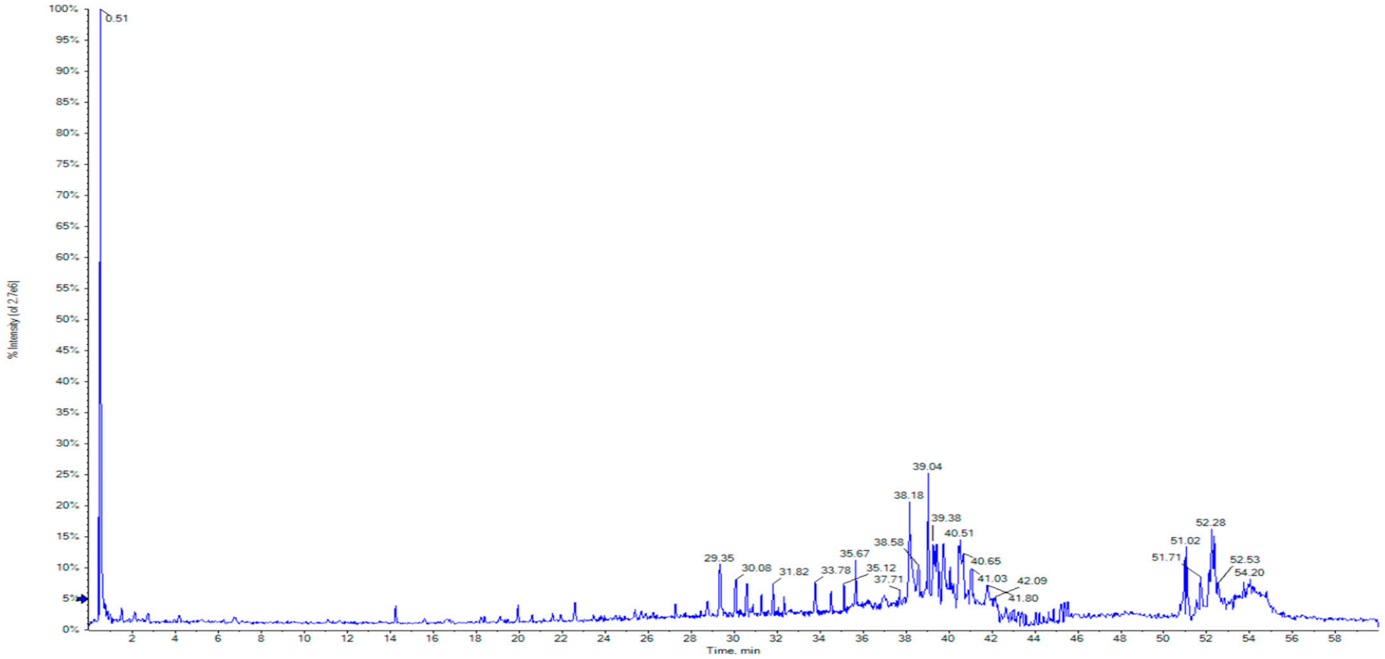

Figure S2. Representative total ion chromatograms (TIC) in negative ESI mode of callus extracts from one sample per species: (A) *G. max* (cv. Nessie), (B) *L. angustifolius* (cv. Bolero), (C) *P. sativum* (cv. Karacter). Data acquired in negative ESI mode using UHPLC–MS/MS, as described in Section 4.5.

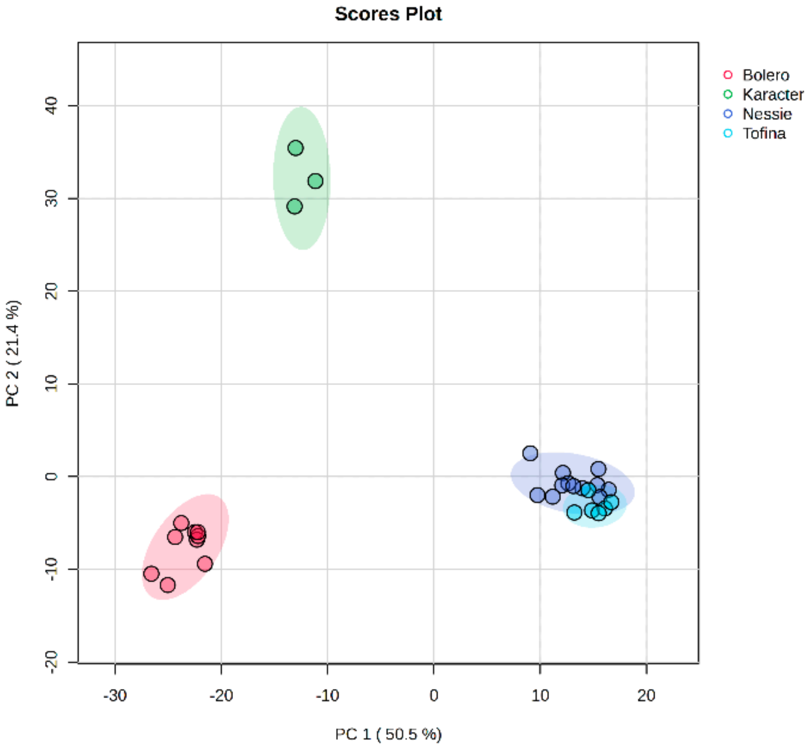

Figure S3. Principal component analysis (PCA) of the 290 curated metabolites showing clear interspecific metabolic differentiation. PC1 and PC2 explained 50.5% and 21.4% of the total variance, respectively. Samples clustered by species, forming distinct, well-separated groups. Ellipses represent the dispersion of each group.

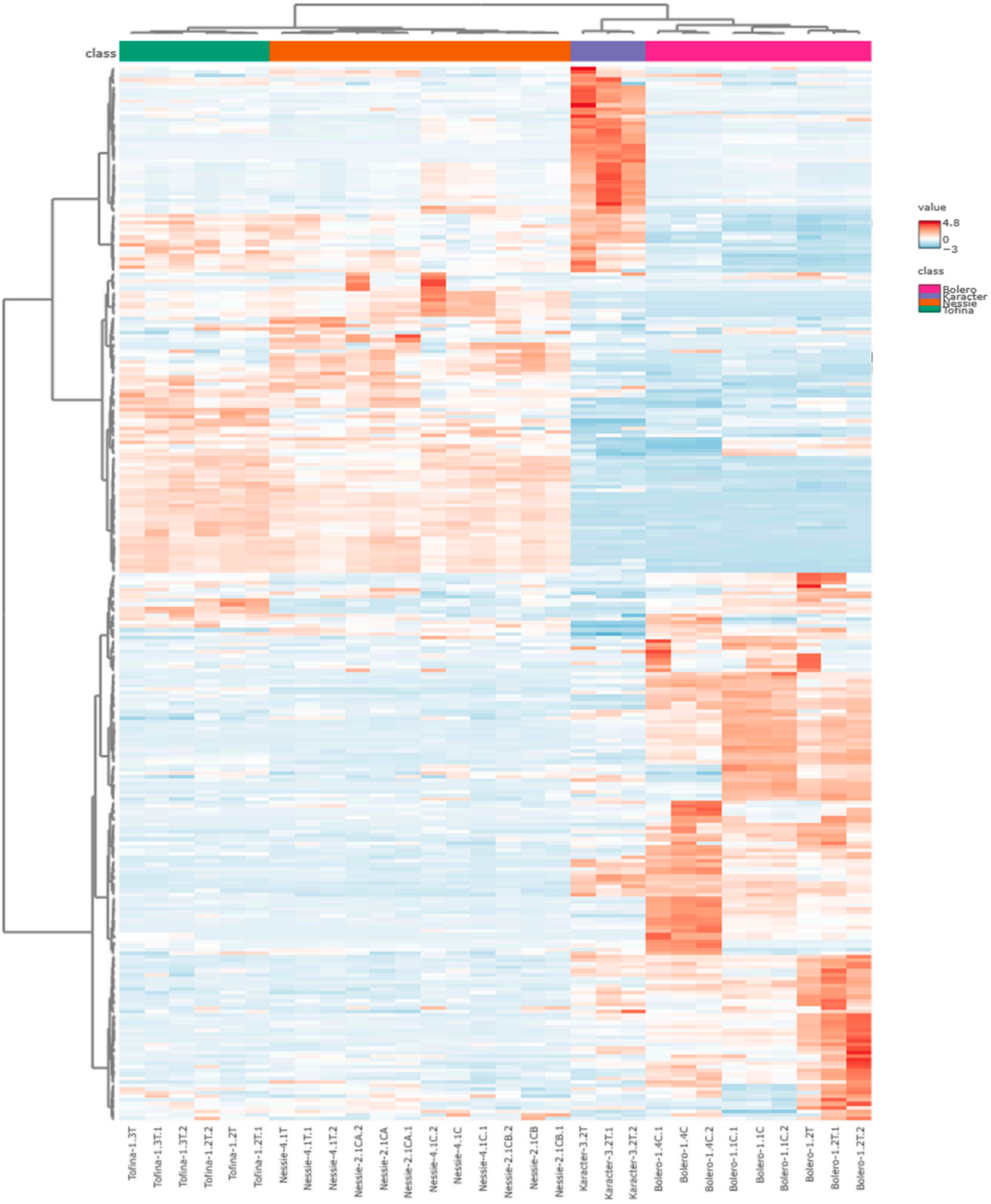

Figure S4. Hierarchical clustering analysis (HCA) of the 290 curated metabolites showing clear species-level metabolic structuring. Unsupervised clustering of samples and metabolites resolved three primary branches corresponding to the three species, with tight grouping of biological replicates within each cluster. Color scale represents normalized relative abundance (red, higher; blue, lower).

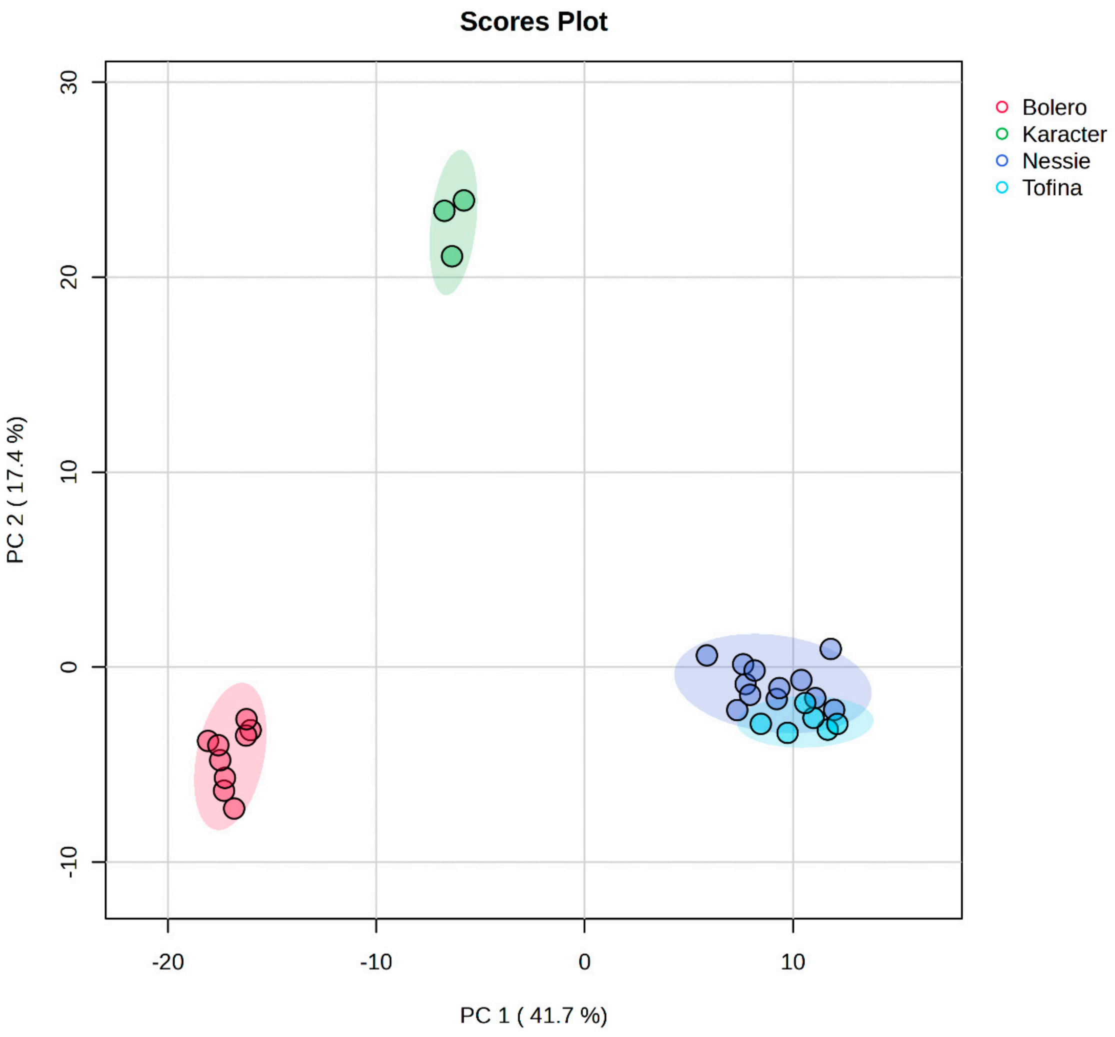

Figure S5. Principal component analysis (PCA) of callus samples based on the specialised metabolite subset (n = 234), excluding primary metabolites (sugars, organic acids, amino acids, nucleotides, and general cellular lipids). The first principal component (PC1) explained 41.7% of the total variance, while the second principal component (PC2) accounted for 17.4%, together capturing 59.1% of the variability of the specialised metabolite dataset.

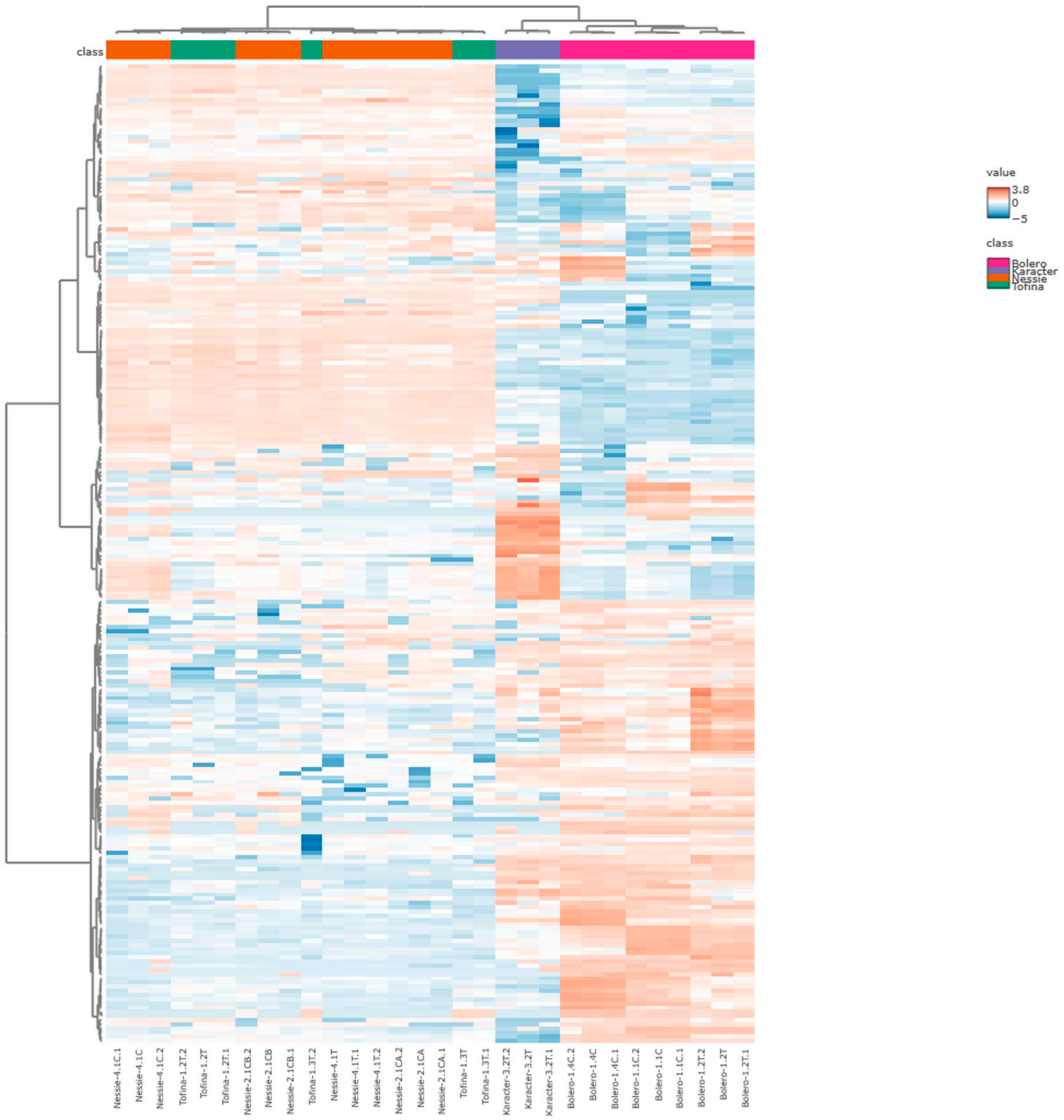

Figure S6. Heatmap and hierarchical clustering analysis (HCA) of callus samples based on the specialised metabolite subset (n = 234), excluding primary metabolites. Each column represents an individual sample, each row an annotated metabolite. Abundances are colour-coded (orange = high, blue = low; mean-centred, unit-variance scaled). Samples are grouped by genotype: Nessie and Tofina (*G. max*, orange and green), Karacter (*P. sativum*, purple), and Bolero (*L. angustifolius*, pink). The dendrogram reveals clear species-level clustering, with the two soybean cultivars grouping together and clearly separated from pea and lupin, in agreement with the corresponding PCA (Figure S4). Distinct species-specific blocks of co-enriched metabolites are visible across the heatmap, supporting the species-level organisation of specialised metabolism in dedifferentiated tissues.

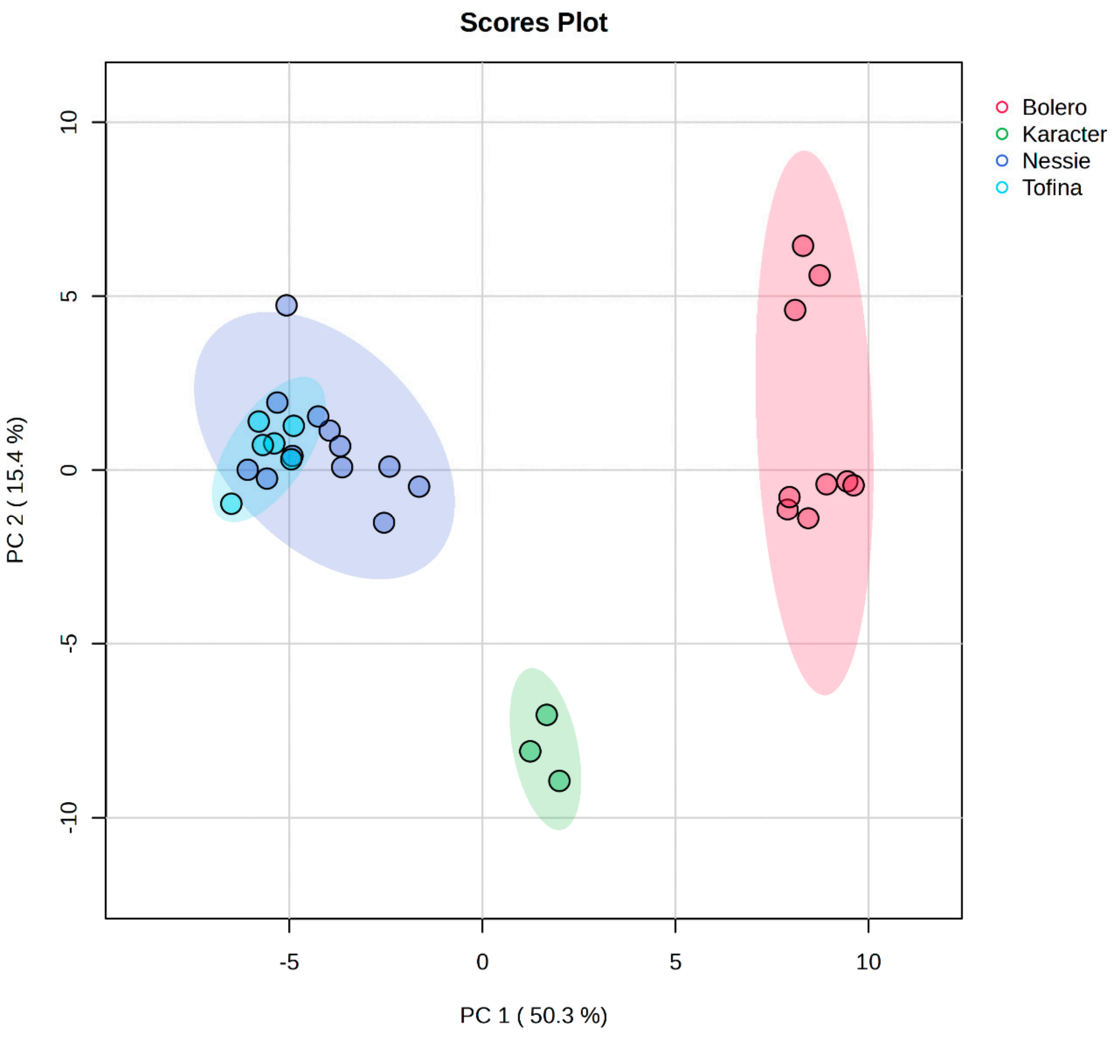

Figure S7. PCA of callus samples based on the saponin metabolite subset (n=38). The first principal component (PC1) explained 50.3% of the total variance, while the second principal component (PC2) accounted for 15.4%, together capturing 65.7% of the variability of the saponin dataset. level clustering, with the two soybean cultivars grouping together and clearly separated from pea and lupin.

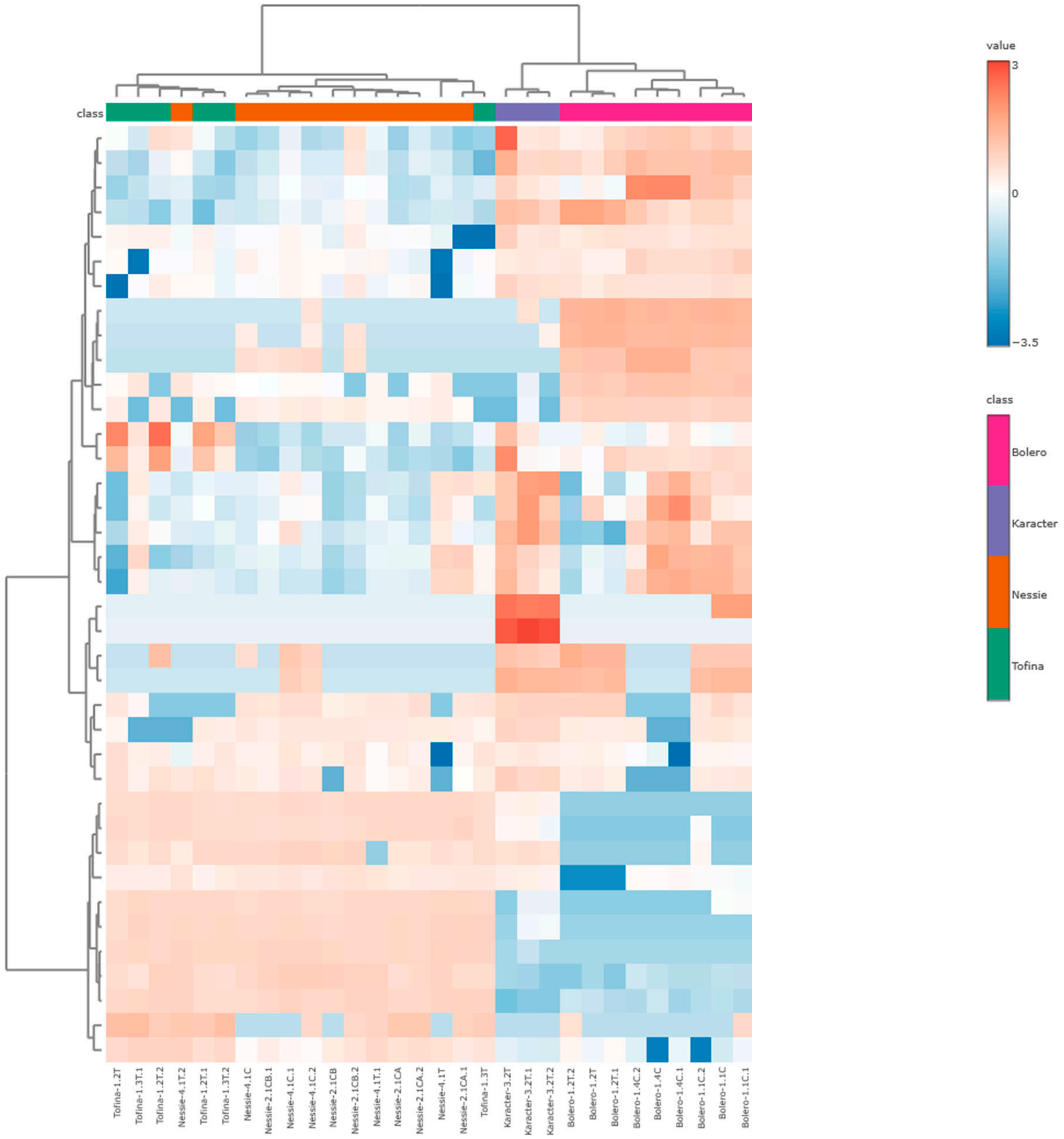

Figure S8. Heatmap and hierarchical clustering analysis (HCA) of callus samples restricted to the saponin subset (n = 38). Each column represents an individual sample, each row an annotated metabolite. Abundances are colour-coded (orange = high, blue = low; mean-centred, unit-variance scaled). Samples are grouped by genotype: Nessie and Tofina (*G. max*, orange and green), Karacter (*P. sativum*, purple), and Bolero (*L. angustifolius*, pink). The dendrogram reveals clear species-level clustering, with distinct species-specific saponin signatures: soybean calli enriched in soyasapogenol B and E glycosides, lupin calli in hederagenin and bayogenin conjugates, and pea calli displaying a distinct saponin block.

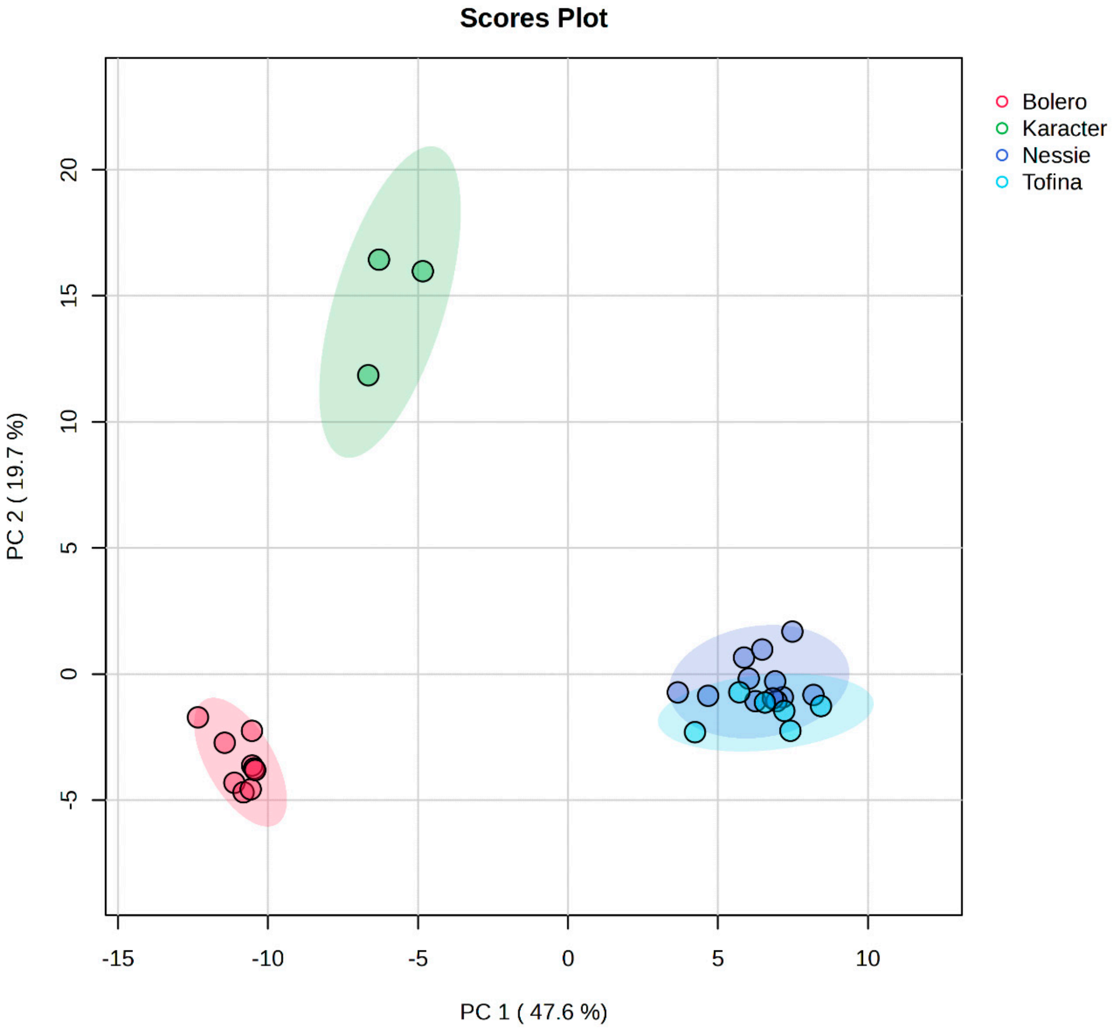

Figure S9. Principal component analysis (PCA) of callus samples restricted to the flavonoid and isoflavonoid metabolite subset (n= 124). The first principal component (PC1) explained 47.6% of the total variance, while the second principal component (PC2) accounted for 19.7%, together capturing 67.3% of the variability of the flavonoid dataset. Each point represents an individual callus sample, color-coded by genotype: Bolero (*Lupinus angustifolius*, red), Karacter (*Pisum sativum*, green), Nessie (*Glycine max*, purple), and Tofina (*Glycine max*, light blue). Three well-separated clusters are observed corresponding to soybean (Nessie and Tofina overlapping), pea (Karacter), and lupin (Bolero), confirming that flavonoid and isoflavonoid profiles are highly species-specific.

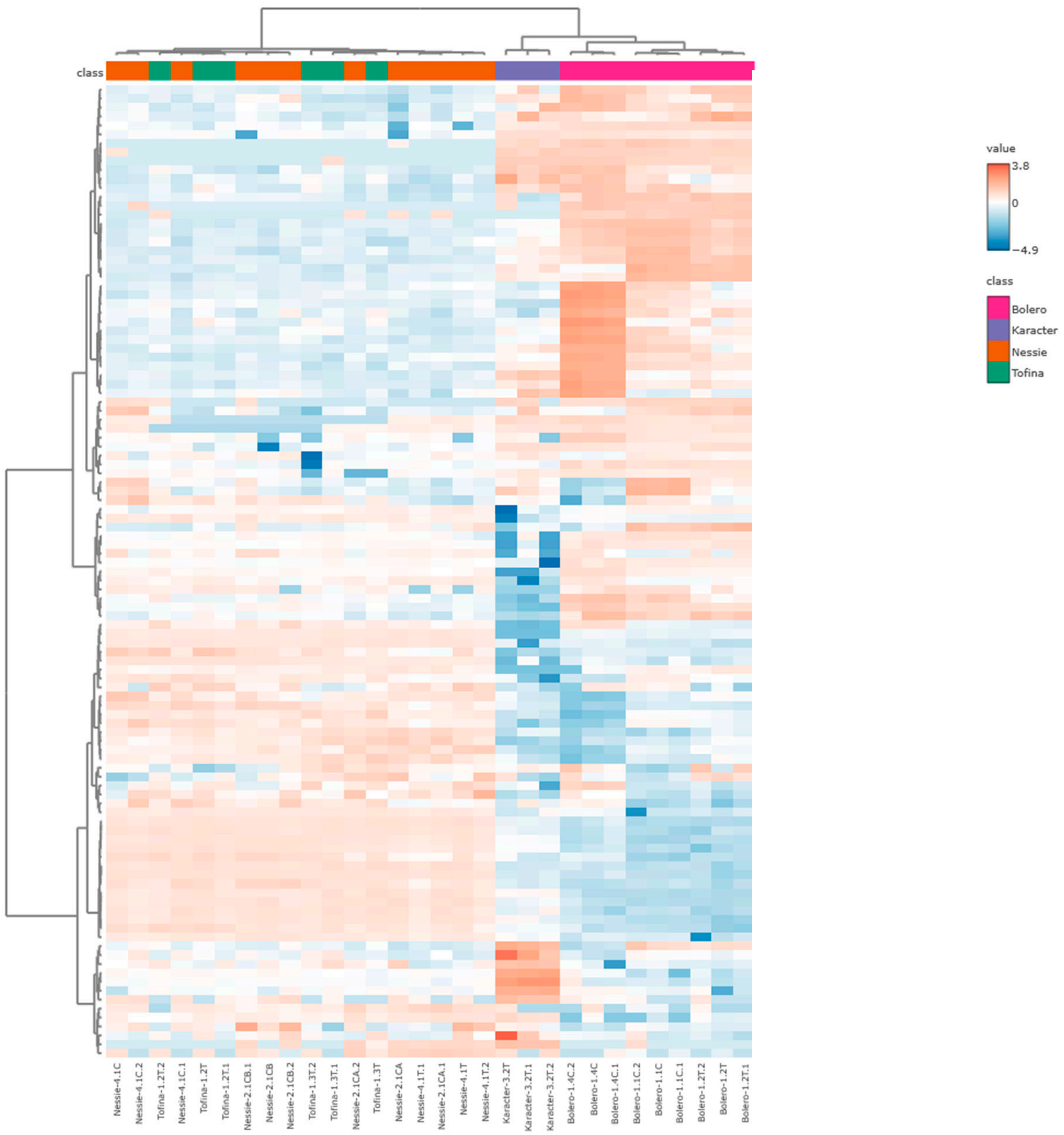

Figure S10. Heatmap and hierarchical clustering analysis (HCA) of callus samples restricted to the flavonoid and isoflavonoid subset. Each column represents an individual sample, each row an annotated metabolite. Abundances are colour-coded (orange = high, blue = low; mean-centred, unit-variance scaled). Samples are grouped by genotype: Nessie and Tofina (*G. max*, orange and green), Karacter (*P. sativum*, purple), and Bolero (*L. angustifolius*, pink). The dendrogram reveals clear species-level clustering, with distinct species-specific signatures: lupin calli enriched in prenylated isoflavonoids, soybean calli in classical isoflavonoid glycosides, and pea calli displaying a distinct block of methylated flavonoid derivatives.

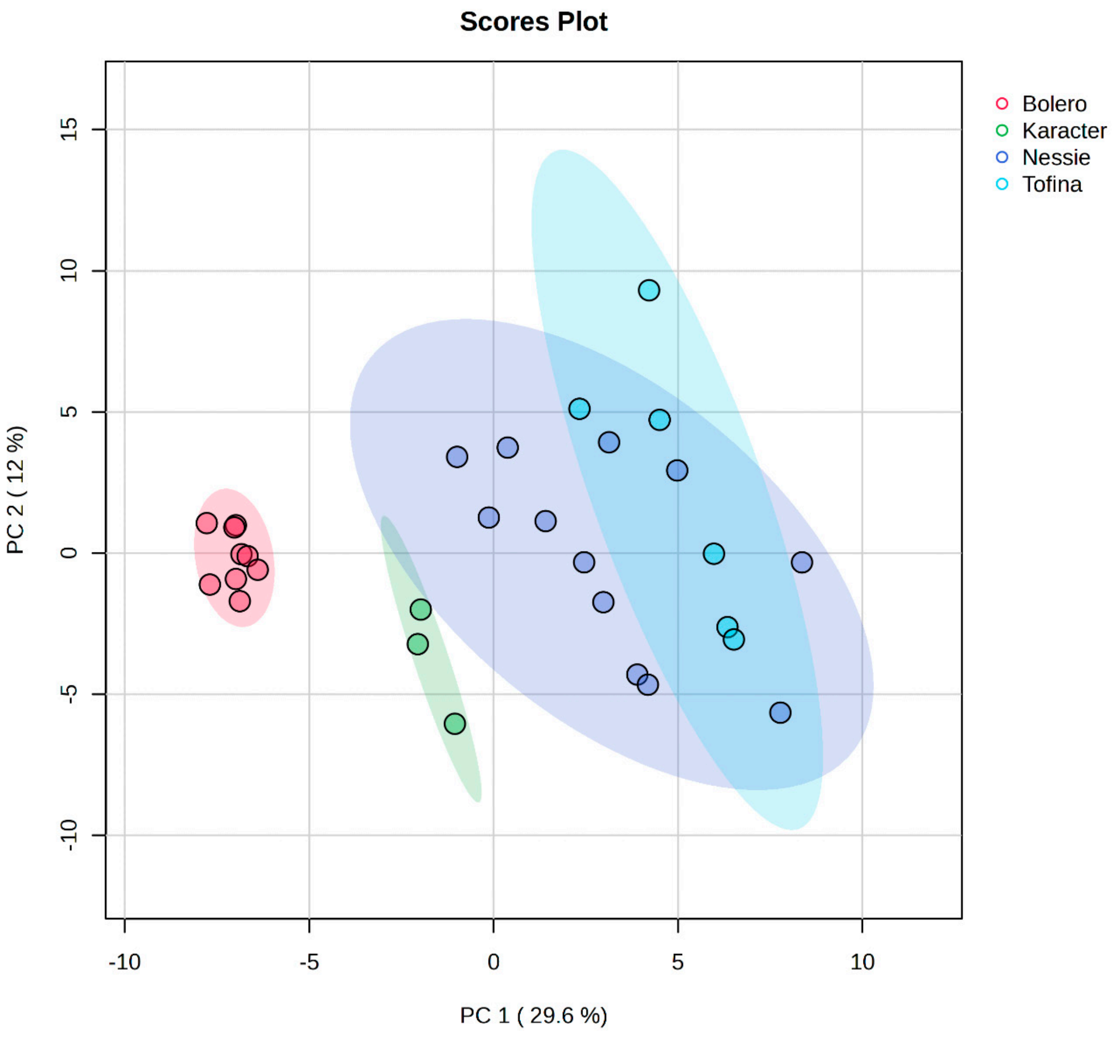

Figure S11. Principal component analysis (PCA) of callus samples restricted to the hydroxycinnamic acid and conjugate metabolite subset (n= 58). PC1 and PC2 explained 29.6% and 12% of the variance, respectively. Each point represents an individual callus sample, color-coded by genotype: Bolero (*Lupinus angustifolius*, red), Karacter (*Pisum sativum*, green), Nessie (*Glycine max*, purple), and Tofina (*Glycine max*, light blue). Lupin and pea calli formed clearly separated clusters, while the two soybean cultivars showed broader within-group dispersion, reflecting greater intraspecific variability in hydroxycinnamic acid composition.

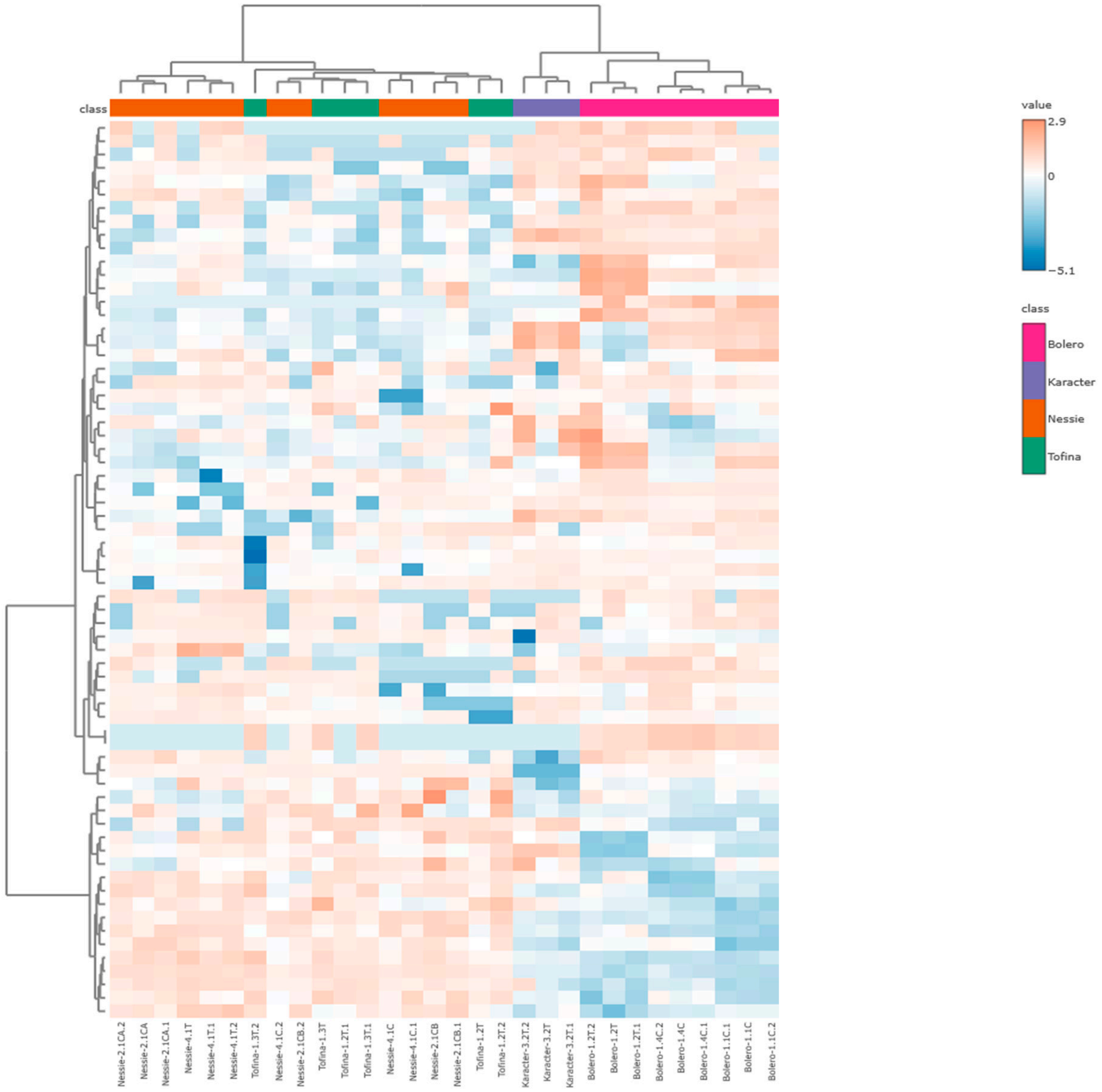

Figure S12. Heatmap and hierarchical clustering analysis (HCA) of callus samples restricted to the hydroxycinnamic acid subset (n = 58). Each column represents an individual sample, each row an annotated metabolite. Abundances are colour-coded (orange = high, blue = low; mean-centred, unit-variance scaled). Samples are grouped by genotype: Nessie and Tofina (*G. max*, orange and green), Karacter (*P. sativum*, purple), and Bolero (*L. angustifolius*, pink). The dendrogram reveals clear species-level clustering, with species-specific blocks of co-enriched hydroxycinnamate conjugates visible across the heatmap.

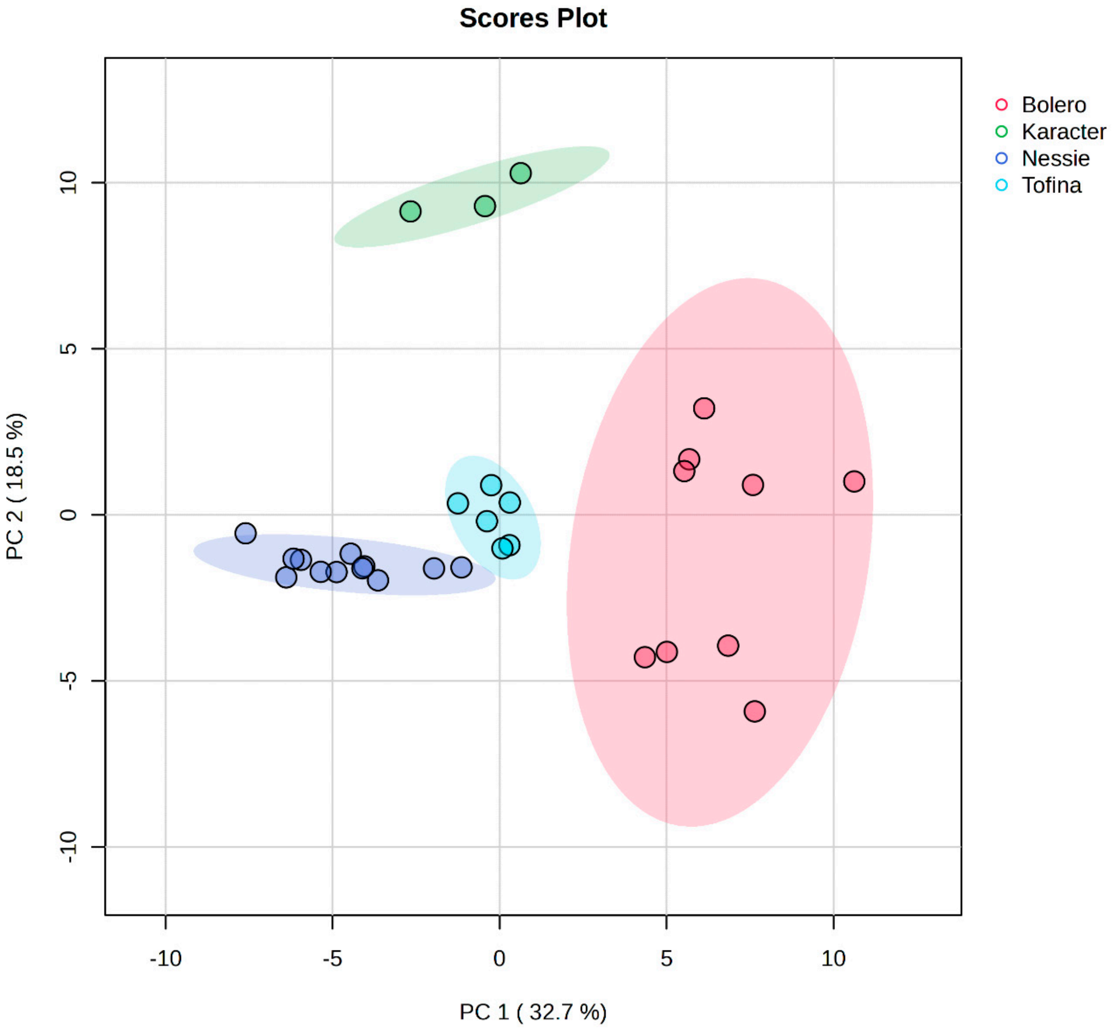

Figure S13. Principal component analysis (PCA) of callus samples restricted to the primary metabolite subset (n=56). comprising sugars, organic acids, amino acids, nucleotides, and general cellular lipids. PC1 and PC2 explained 32.7% and 18.5% of the variance, respectively. Each point represents an individual callus sample, color-coded by genotype: Bolero (*Lupinus angustifolius*, red), Karacter (*Pisum sativum*, green), Nessie (*Glycine max*, dark blue), and Tofina (*Glycine max*, light blue). Species-level grouping is observed but with greater within-species dispersion than for specialised metabolites, and a partial separation between Nessie and Tofina cultivars becomes visible along PC1, suggesting that primary metabolite profiles capture physiological differences among cultivars in addition to species-level distinctions.

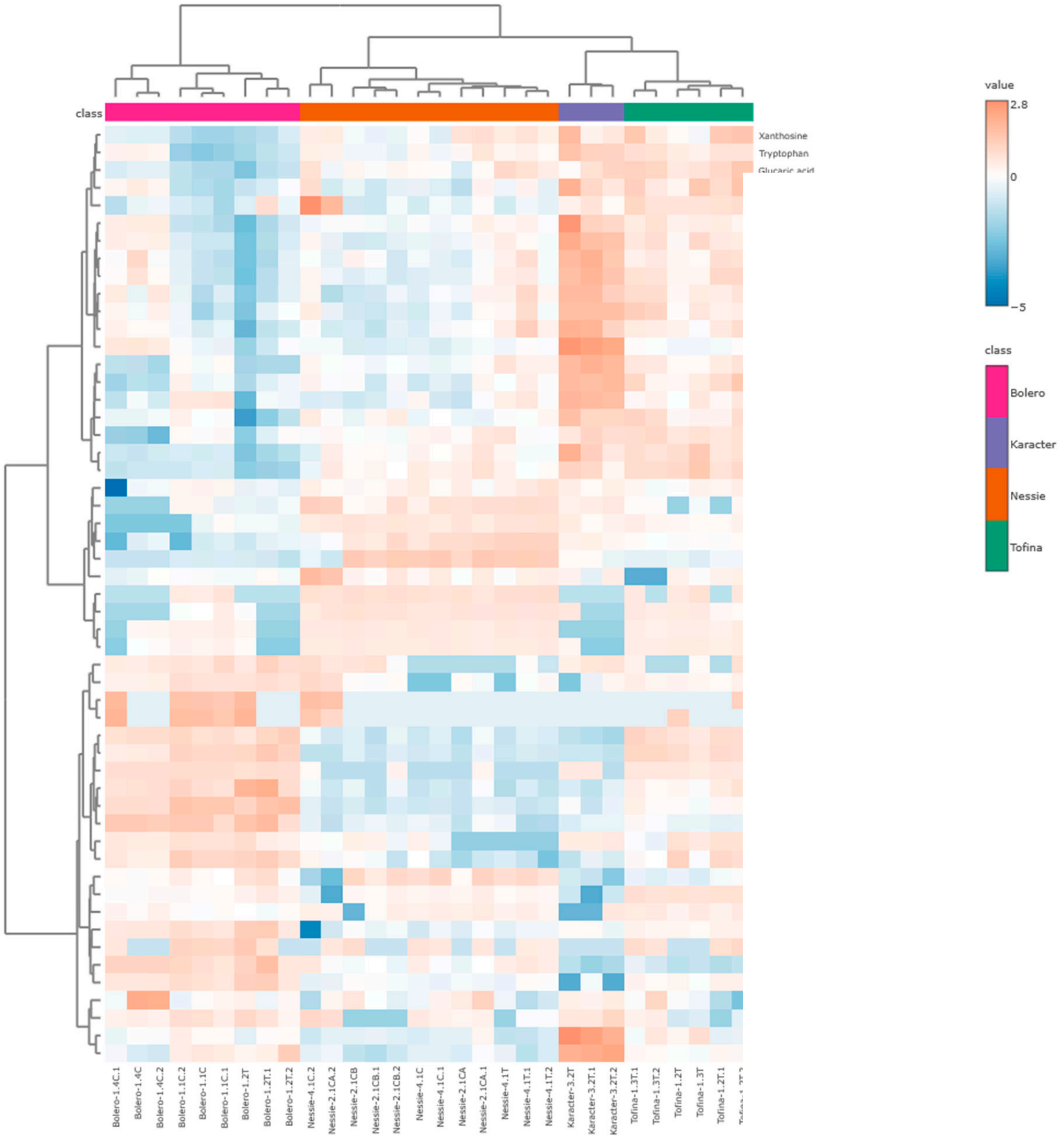

Figure S14. Heatmap and hierarchical clustering analysis (HCA) of callus samples restricted to the primary metabolite subset (n = 56). Each column represents an individual sample, each row an annotated metabolite. Abundances are colour-coded (orange = high, blue = low; mean-centred, unit-variance scaled). Samples are grouped by genotype: Bolero (*L. angustifolius*, pink), Nessie (*G. max*, orange), Karacter (*P. sativum*, purple), and Tofina (*G. max*, green). Species-level clustering is observed, with the two soybean cultivars forming distinct sub-groups within the soybean clade, indicating that primary metabolite profiles capture physiological differences between cultivars in addition to species-level distinctions.

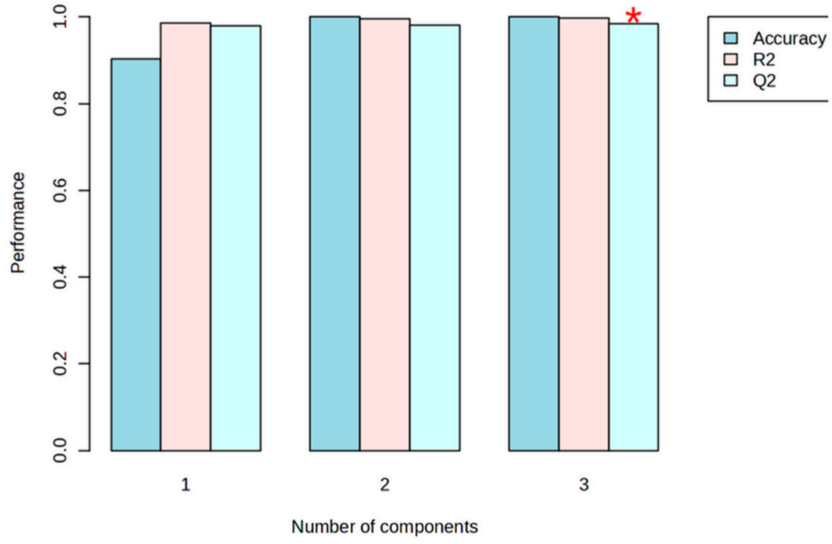

Figure S15. PLS-DA cross-validation performance. Leave-one-out cross-validation performance of the PLS-DA model as a function of the number of latent variables (1 to 3). For each number of components, classification accuracy (blue), cumulative goodness-of-fit R^2 (pink), and cross-validated predictive ability Q^2 (light blue) are shown. The optimal model (red asterisk) was obtained with 3 latent variables (Accuracy = 1.000, R^2 = 0.998, Q^2 = 0.987).

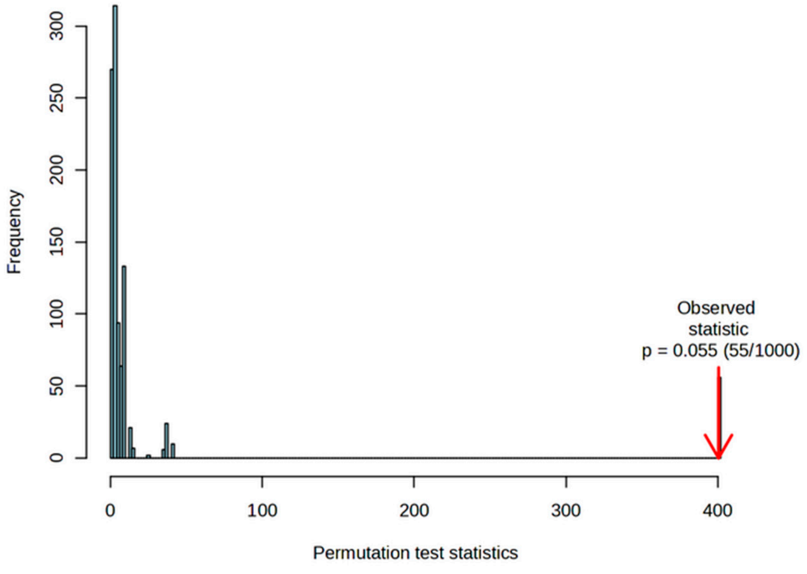

Figure S16. PLS-DA permutation test. Histogram of test statistic values obtained by random permutation of class labels (1000 permutations) compared with the observed test statistic from the actual PLS-DA model (red arrow, ~400). Prediction accuracy was used as the test statistic. The empirical p-value was 0.055 (55/1000 permutations), bounded by the limited number of replicates per class as noted in the MetaboAnalyst documentation for small-sample datasets.

Supplementary Note S1. Untargeted metabolomic data-processing and filtering workflow.

Raw UHPLC–MS/MS data were imported into Progenesis QI v2.3 (Nonlinear Dynamics, Waters, Newcastle, UK) for peak picking, alignment, and normalisation across samples. Peak picking and alignment yielded a total of 36,440 m/z features detected across all biological samples and quality control (QC) injections. Features were subsequently filtered based on statistical criteria (one-way ANOVA $p < 0.05$ and fold change > 2 between species groups), retaining 10,638 features for tentative annotation. Tentative annotation was performed by accurate-mass matching against internal and external metabolite databases and the relevant literature, augmented by manual MS/MS-based structural elucidation guided by characteristic fragmentation patterns of isoflavonoids, flavonoids, hydroxycinnamic acid conjugates, and triterpenoid saponins, in both negative (primary) and positive (confirmatory) ESI modes. Manual curation of the annotated features included the removal of redundant entries corresponding to dimers, adducts, and in-source fragments identified during compound-by-compound MS/MS review. This curation step yielded a final non-redundant curated dataset of 290 compounds, which was used for all downstream multivariate analyses (PCA, HCA, PLS-DA), and per-compound-class subset analyses) reported in the main manuscript.
