# Supplementary material for: Species-Specific Metabolic Identities Persist in Fabaceae Callus Cultures Under Standardized Culture Conditions
Source: Plants (Basel). 2026 Jul 27;15(15):2299. doi: 10.3390/plants15152299 (PMC13468598; doi:10.3390/plants15152299)
Supplement: Supplementary file 1 [file plants-15-02299-s001.zip › Supplementary Table S1.pdf]

Table S1. Summary of the callus induction attempts 60 days after induction across all Fabaceae species, cultivars, and explant types tested in this study.

Species	Variety	Explant	Code	Callus aspect
Soybean	Nessie	Cotyledon	2.1CA	Vigorous proliferation; friable, well-developed callus
			2.1CB	Vigorous proliferation; friable, well-developed callus
			4.1C	Vigorous proliferation; friable, well-developed callus
			2.2C	Vigorous proliferation; friable, well-developed callus
		Epicotyl	4.2T	Vigorous proliferation; friable, well-developed callus
			4.1T	Vigorous proliferation; friable, well-developed callus
			2.1T	Vigorous proliferation; friable, well-developed callus
	Tofina	Epicotyl	1.2T	Vigorous proliferation; friable, well-developed callus
			3.1T	Vigorous proliferation; friable, well-developed callus
			1.1T	Vigorous proliferation; friable, well-developed callus
Blue lupin	Bolero	Cotyledon	3.1C	Limited proliferation
			1.1C	Vigorous proliferation; friable, well-developed callus
			1.4C	Vigorous proliferation; friable, well-developed callus
			2.2C	Active proliferation; friable callus
		Epicotyl	4.2T	No detectable proliferation
			5.1T	No detectable proliferation
			1.1T	No detectable proliferation
			1.2T	Active proliferation; friable callus
Pea	Karacter	Cotyledon	6.1C	No detectable proliferation
		Epicotyl	3.1T	No detectable proliferation
			3.2T	Proliferation arrest
			6.2T	No detectable proliferation
			3.2T	limited proliferation
			5.1T	No detectable proliferation
	Greenway	Epicotyl	6.2T	Very slow biomass accumulation
			1.2T	Very slow biomass accumulation
			4.1T	Very slow biomass accumulation
Faba bean	Magnolia	Cotyledon	2.1C	No detectable proliferation
			2.2C	No detectable proliferation
		Epicotyl	4.2T	No detectable proliferation
			4.1T	No detectable proliferation
			2.1T	No detectable proliferation
			2.2T	No detectable proliferation
	Jasmin	Epicotyl	3.2T	No detectable proliferation
Chickpea	Elmo	Epicotyl	1.1T	No detectable proliferation
			4.1T	No detectable proliferation
			4.2T	No detectable proliferation
	Orion	Epicotyl	2.1T	No detectable proliferation
			2.2T	No detectable proliferation

*Callus induction attempts were performed under identical hormonal conditions for five Fabaceae species, soybean (*Glycine max*; cultivars Nessie and Tofina), lupin (*Lupinus angustifolius*; cv. Bolero), pea (*Pisum sativum*; cultivars Karacter and Greenway), faba bean (*Vicia faba*; cultivars Magnolia and Jasmin), and chickpea (*Cicer arietinum*; cultivars Elmo and Orion). For each line, the species, cultivar, explant type, line code, and morphological/proliferative characteristics are reported. Morphological/proliferative descriptors are based on visual assessment during routine subculture. Only soybean, lupin, and pea callus lines indicated in red proliferated stably under the applied hormonal conditions were retained for the metabolomic analyses presented in this study. The remaining species and cultivars are reported here to document the full scope of the induction screening.*
